# Propranolol improves cognitive function and glymphatic system recovery through STAT3/MMP2 pathway after TBI

**DOI:** 10.1371/journal.pone.0353852

**Published:** 2026-08-21

**Authors:** Dongming Gao, Guohui Huang, Lei Li, Peng Yu

**Affiliations:** 1 Department of Neurosurgery, Shanghai Tenth People’s Hospital, Tongji University, Shanghai, China; 2 Department of Neurosurgery, Huadong Hospital, Fudan University, Shanghai, China; University of Florida, UNITED STATES OF AMERICA

## Abstract

There is no effective treatment for cognitive impairment after traumatic brain injury (TBI). Previous studies have demonstrated that propranolol may improve cognitive function. The glymphatic system plays a crucial role in cognitive impairment after TBI. Thus, we explored the mechanism by which propranolol improves cognitive function and promotes glymphatic system recovery after TBI. In vivo, rats were subjected to controlled cortical impact (CCI) and treated with saline, propranolol, S31-201 (an inhibitor of STAT3) and SB-3CT (an inhibitor of MMP2) respectively. Rats then underwent a battery of cognitive tests. We also assessed white matter (WM) integrity and glymphatic system function. The expression of phosphorylated tau (p-tau), amyloid-β (Aβ), aquaporin 4 (AQP4), signal transducer and activator of transcription 3 (STAT3), phosphorylated STAT3 (p-STAT3), and matrix metalloproteinase-2 (MMP2) was evaluated by immunocytochemistry and Western blot. In vitro, the levels of STAT3, p-STAT3, MMP2 and AQP4 were assessed after astrocytes were treated with propranolol, noradrenaline, S31-201 and SB-3CT respectively. In vivo results showed that propranolol, S31-201 and SB-3CT improved cognitive function and restored glymphatic system function after TBI. The deposition of p-tau and Aβ was decreased, and WM integrity was restored in TBI rats treated with propranolol, S31-201 and SB-3CT. Propranolol, S31-201 and SB-3CT induced the overexpression of AQP4 in blood vessels and astrocytes after TBI. In addition, the levels of p-STAT3 and MMP2 were knocked down in propranolol-treated TBI rats. In vitro, propranolol reversed the upregulated expression of p-STAT3 and MMP2 in noradrenaline-treated astrocytes. The noradrenaline-induced impairment of AQP4 expression in astrocytes was antagonized by propranolol, S31-201 and SB-3CT. In conclusion, propranolol may improve cognitive function and facilitate glymphatic system functional recovery after TBI through the STAT3/MMP2 pathway.

## Introduction

Traumatic brain injury (TBI) induces multiple symptoms, such as fatigue, headaches, anxiety and cognitive impairment [1]. However, there is still no effective treatment for TBI-induced cognitive impairment. Therefore, it is urgent to explore novel therapeutic strategies for this condition. The glymphatic system is a cerebral waste clearance pathway that facilitates the removal of metabolic wastes and toxins from the brain. Growing evidence indicates that impairment of the glymphatic system contributes to post-TBI neurological disorders by promoting tau/amyloid-β (Aβ) deposition [2, 3]. Thus, we hypothesized that restoration of the glymphatic system could improve cognitive function after TBI.

As a β-blocker, propranolol reduces mortality and improves long-term functional outcomes after TBI by blocking excessive catecholamines [4]. Norepinephrine (NE), a neuromodulator and catecholamine, regulates the activity of neuronal and non-neuronal cells, including astrocytes, neurons and microglia. Microglial and astrocytic activation promotes Aβ deposition, which further induces neuronal death in Alzheimer’s disease and post-TBI cognitive impairment [5, 6]. In addition, previous studies have shown that norepinephrine reduces the clearance efficiency of the glymphatic system [7]. Therefore, propranolol may interact with the glymphatic system through modulating neuronal and non-neuronal cells.

Aquaporin 4 (AQP4), a water channel protein predominantly localized in astrocytic endfeet surrounding capillaries, plays an essential role in the glymphatic system [8]. In Alzheimer’s disease, disruption of AQP4 leads to impaired glymphatic function and accelerates the accumulation of neurotoxins and metabolic wastes [9].

Accumulating evidence indicates that AQP4 can be regulated by matrix metalloproteinase-2 (MMP2) via multiple mechanisms [10]. Meanwhile, norepinephrine modulates MMP2 expression by activating the activator of transcription 3 (STAT3) signaling pathway [11]. Thus, we hypothesized that propranolol could restore cognitive function and glymphatic system function in TBI patients through regulating the STAT3/MMP2 pathway.

## Materials and methods

### Animals and TBI model

All experiments were performed in accordance with the guidelines approved by the Animal Care and Use Committee of Tongji University. Male Sprague-Dawley rats (12 weeks old, body weight 280–320 g) were randomly assigned to five groups: TBI group, TBI + propranolol group, sham group, TBI + S31-201 (an inhibitor of STAT3) group and TBI + SB-3CT (an inhibitor of MMP2) group. Propranolol (4 mg/kg) was dissolved in phosphate-buffered saline (PBS) and administered once daily for three consecutive days after surgery [12]. S31-201 (5 mg/kg, i.p.) was administered 30 min before surgery [13]. SB-3CT (50 mg/kg, dissolved in 10% DMSO) was administered intraperitoneally 30 min after TBI, with subsequent injections at 6 h and 12 h post-injury [14].

In this study, TBI rats (n = 6 per group) were anesthetized with chloral hydrate (3.0 mL/kg, i.p.) to relieve pain. During surgery, the animals breathed spontaneously with 1.5% isoflurane delivered in a 2:1 N_2_O/O_2_ mixture via a facemask connected to a modified FLUOTEC 3 Vaporizer (Fraser Harlake, Orchard Park, NY). Rectal temperature was maintained at 37 °C throughout the procedure. Surgical procedures and moderate controlled cortical impact (CCI) injury were performed as previously described [15]. Seven days after injury, the rats were re-anesthetized with chloral hydrate and then euthanized for subsequent experiments.

### Cell culture

Primary astrocytes were isolated from the cerebral cortex of neonatal rats within 24h after birth. Cerebral cortices were dissected and transferred into sterile tubes. After incubation with 0.25% trypsin (Sigma, St. Louis, MO, USA) for 10 min at 37 °C, the tissue was centrifuged at 1000 rpm for 5 min. The cell pellet was resuspended in Dulbecco’s modified Eagle’s medium (DMEM) supplemented with 10% heat-inactivated fetal bovine serum (FBS, Sigma, USA). The cell suspension was seeded into culture dishes for adhesion and cultivation. After 10 days of culture, microglia and oligodendrocytes were eliminated by orbital shaking at 200 rpm for 2 h at 37 °C[16]. When primary astrocytes reached 70%–90% confluency in 6-well plates, the cells were cultured for another 24 h and then divided into six groups: control group, norepinephrine (50 µM) group, propranolol (50 µM) group, norepinephrine (50 µM) + propranolol (50 µM) group, norepinephrine (50 µM) + S31-201 (10 µM) group, and norepinephrine (50 µM) + SB-3CT (25 µM) group [17–19]. After 24 h of treatment, the cells were harvested for subsequent experiments

### Behavioral assessments

Novel object recognition (NOR) was performed to evaluate short-term memory and visual learning. The test relies on animals’ natural tendency to investigate unfamiliar objects, with a 4-hour retention interval. The NOR index, defined as the percentage of time spent exploring the novel object, was recorded over a 5-minute test period.

The odor preference test was used to assess long-term memory, based on animals’ preference for novel odors. We calculated the percentage of time animals spent investigating the two wooden beads with unfamiliar odors.

The Morris water maze (MWM) test, which evaluates spatial and visual learning and memory, was used to detect hippocampal memory impairments. We recorded escape latency (time to reach the platform) and the percentage of time animals spent swimming in each quadrant [20, 21].

### Histology and immunohistochemistry

After the rats were decapitated, brain tissues were dissected and fixed in buffered formaldehyde. A series of 6 μm sections were cut from paraffin blocks. The sections were incubated overnight at 4 °C with primary antibodies against phosphorylated tau (p-tau) (1:200, Abcam, Cambridge, UK) and Aβ (1:100, Abcam, Cambridge, UK) [20]. Subsequently, the sections were incubated with the secondary antibody (1:100, Abcam, Cambridge, UK) for 30 minutes. Each immunostained section was imaged with a 40 × objective (Olympus BX40; Tokyo, Japan) using the MCID image analysis system (Imaging Research, St. Catharines, Ontario, Canada). The total number of immunopositive cells was counted in each section.

Luxol Fast Blue (LFB; Sigma-Aldrich, USA) staining was applied to assess myelin density in brain tissues [20].

### Glymphatic system measurement

Glymphatic system function was assessed using a previously established protocol. Anesthetized rats were secured in a stereotaxic frame, and a 30 GA needle was inserted into the cisterna magna to ensure uniform distribution of dextran. A total volume of 5 μL dextran (Sigma, MW: 70 kDa, USA) was delivered into the cisterna magna of TBI rats over 5 min via a syringe pump at 2 μL/min. One hour after the start of infusion, the rats were euthanized [20,22]. Animals were transcardially perfused with 0.9% saline and subsequently with 4% paraformaldehyde. Coronal sections of 80 μm thickness were prepared and imaged under a 10 × laser-scanning confocal microscope (Zeiss LSM 510 NLO, Carl Zeiss, Germany). The MCID image analysis system (Imaging Research, St. Catharines, Ontario, Canada) was applied to quantify fluorescence intensity. Results were expressed as the percentage of fluorescence-positive area in brain cross-sections.

### Fluorescence staining

Rats were euthanized, and serial sections of 6 μm thickness were prepared. After rinsing with distilled water, the sections were incubated overnight at 4 ℃ with primary antibodies targeting FITC (fluorescein isothiocyanate)-conjugated AQP4 (1:200, Abcam, Cambridgeshire, UK), von Willebrand factor (vWF, 1:300, Abcam, Cambridgeshire, UK) and glial fibrillary acidic protein (GFAP, 1:200, Santa Cruz, USA) respectively. On the following day, the sections were incubated with the corresponding secondary antibody (1:100, Abcam, Cambridgeshire, UK) for 30 minutes. All slides were observed and imaged under an Olympus BX40 fluorescence microscope (Tokyo, Japan). In parallel, isolated astrocytes were rinsed with PBS and labelled with FITC-conjugated AQP4 antibody (1:200, Abcam, Cambridgeshire, UK). Cell nuclei were counterstained with DAPI [23].

### Western blot

Briefly, equal volumes of protein samples were separated by SDS-PAGE and transferred onto polyvinylidene difluoride (PVDF) membranes (Millipore, Billerica, MA, USA). After blocking, the membranes were incubated overnight at 4 °C with primary antibodies against MMP2 (1:2000, Abcam, Cambridgeshire, UK), STAT3 (1:1000, Abcam, Cambridgeshire, UK), phosphorylated STAT3 (p-STAT3, 1:5000, Abcam, Cambridgeshire, UK), and GAPDH (1:5000, Solarbio, Beijing, China; 1:2000, Abcam, Cambridgeshire, UK), which served as an internal reference. Subsequently, the membranes were incubated with horseradish peroxidase (HRP)-linked secondary antibodies (1:1500; Santa Cruz Biotechnology, USA) for 2 h at room temperature. Protein bands were visualized using SuperSignal West Pico Chemiluminescent Substrate (Pierce, Rockford, IL, USA). Band intensity was measured with Image J software (NIH), and the relative expression levels were normalized to GAPDH [23].

### Statistical analysis

All quantitative measurements were conducted by an investigator who was blinded to group assignments. For each antibody, five slides were obtained from each brain sample, and eight visual fields were captured per slide for statistical analysis. Positively stained areas and the number of immunopositive cells were quantified using the densitometry tool of the MCID image analysis system (Imaging Research, Canada). A unified density threshold, which was higher than the signal of unstained regions, was applied to all experimental groups. The Student’s t-test was used for pairwise comparisons of cognitive function and histological results between groups. All data are expressed as mean ± standard error (SE).

## Result

### Propranolol improved cognitive function after TBI

**Fig 1:**
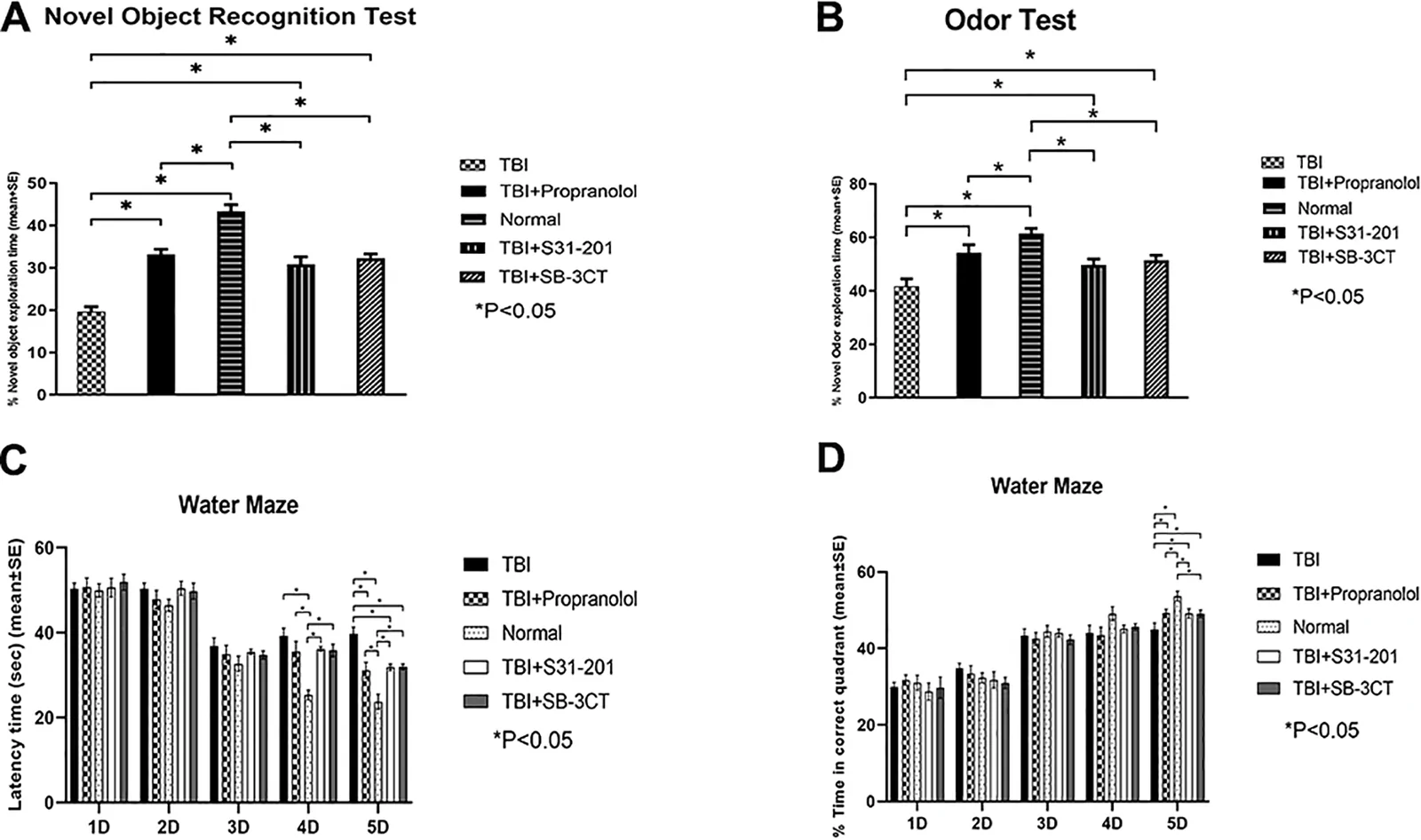
Cognitive function tests in rats. (A) The novel object recognition test revealed markedly improved short-term memory in the propranolol, S31-201 and SB-3CT treatment groups compared with the TBI group. Nevertheless, none of the three treatments fully rescued TBI-induced short-term memory deficits compared with normal rats. (B) The odor test demonstrated obvious long-term memory improvements in the propranolol, S31-201 and SB-3CT treatment groups compared with the TBI group, whereas complete reversal of long-term memory impairment was not achieved relative to normal rats. (C, D) In the Morris water maze test, TBI rats receiving propranolol, S31-201 or SB-3CT displayed significantly ameliorated spatial learning and memory capacities. Still, none of the interventions fully restored spatial cognitive function compared with normal controls. (*p < 0.05, n = 6 per group).

Compared with untreated TBI rats, propranolol, S31-201 and SB-3CT all significantly relieved short- and long-term memory deficits in the novel object recognition and odor tests, though none of the three agents fully recovered memory function compared with the normal rats. In the Morris water maze test, propranolol, S31-201, and SB-3CT all alleviated the deficits in spatial learning and memory in TBI rats. However, none of these three agents fully restored spatial cognitive function compared with the normal rats (Fig 1).

### Propranolol decreased p-tau/Aβ deposition and white matter (WM) damage

To explore the underlying mechanism by which propranolol improves cognitive function, we examined cerebral p-tau and Aβ deposition levels. The results demonstrated that propranolol, S31-201, and SB-3CT all totally reduced TBI-triggered p-tau and Aβ accumulation in the brain. Moreover, no statistically significant differences were detected among the three treatment groups and the normal group.

**Fig 2:**
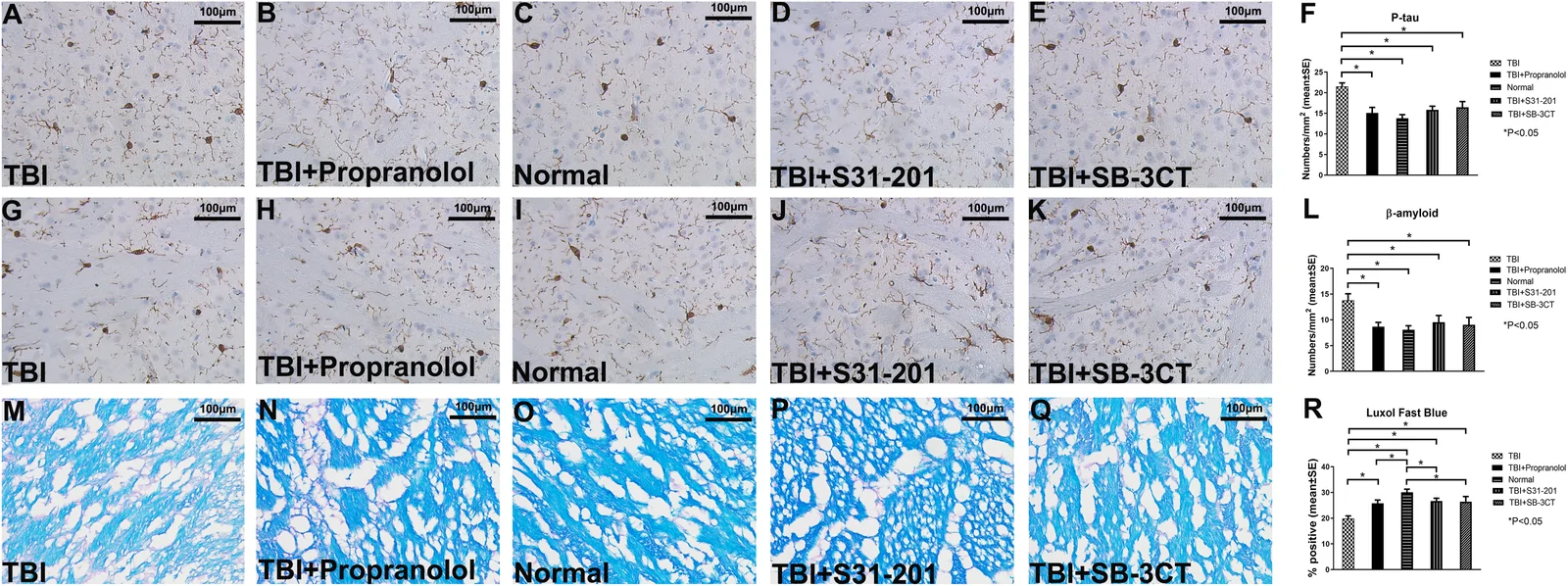
Expressions of p-tau, Aβ and myelin integrity in rat brains. (A-F) Propranolol, S31-201, and SB-3CT all totally alleviated TBI-induced cerebral p-tau accumulation. No statistically significant differences were observed among the three treatment groups and the normal control group. (G–L) Similarly, propranolol, S31-201, and SB-3CT totally lowered TBI-triggered brain Aβ accumulation, with no significant differences detected among the treatment groups and normal controls. (M–R) Myelin density was significantly recovered in drug-treated rats compared with untreated TBI rats. However, none of the three interventions restored myelin density completely to the level of normal rats. (*p < 0.05, n = 6/group,10 × 40 objective lens).

To further verify whether propranolol, S31-201, and SB-3CT could rescue TBI-induced white matter (WM) integrity damage, brain myelin density was quantified. As illustrated in Fig 2, myelin density was markedly restored in rats treated with propranolol, S31-201, or SB-3CT relative to untreated TBI rats. Nevertheless, none of the three interventions fully restored myelin density to the level observed in normal rats (Fig 2).

### Propranolol recovered glymphatic dysfunction in TBI rats

**Fig 3:**
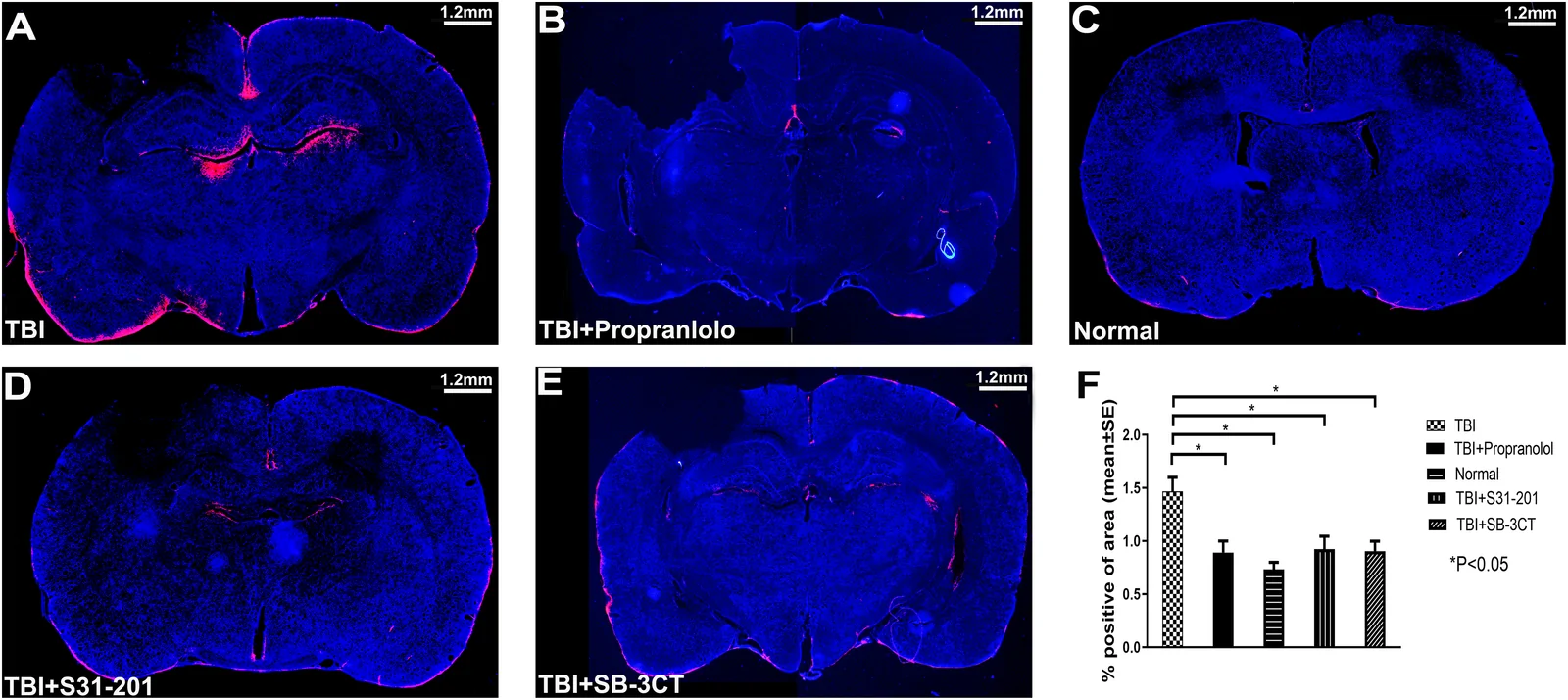
Glymphatic function in rat Brains. (A-F) Propranolol, S31-201 and SB-3CT totally rescued TBI-induced delay in metabolic waste clearance. No significant differences were detected among the three treatment groups and the normal group. (*p < 0.05, n = 5/group).

Since propranolol was found to reduce cerebral p-tau and Aβ deposition following TBI, we further examined glymphatic system function. The results revealed that propranolol, S31-201, and SB-3CT totally rescued TBI-induced delay in metabolic waste clearance. No significant differences were detected among the three treatment groups and the normal group (Fig 3).

### Propranolol restored AQP4 expression after TBI

**Fig 4:**
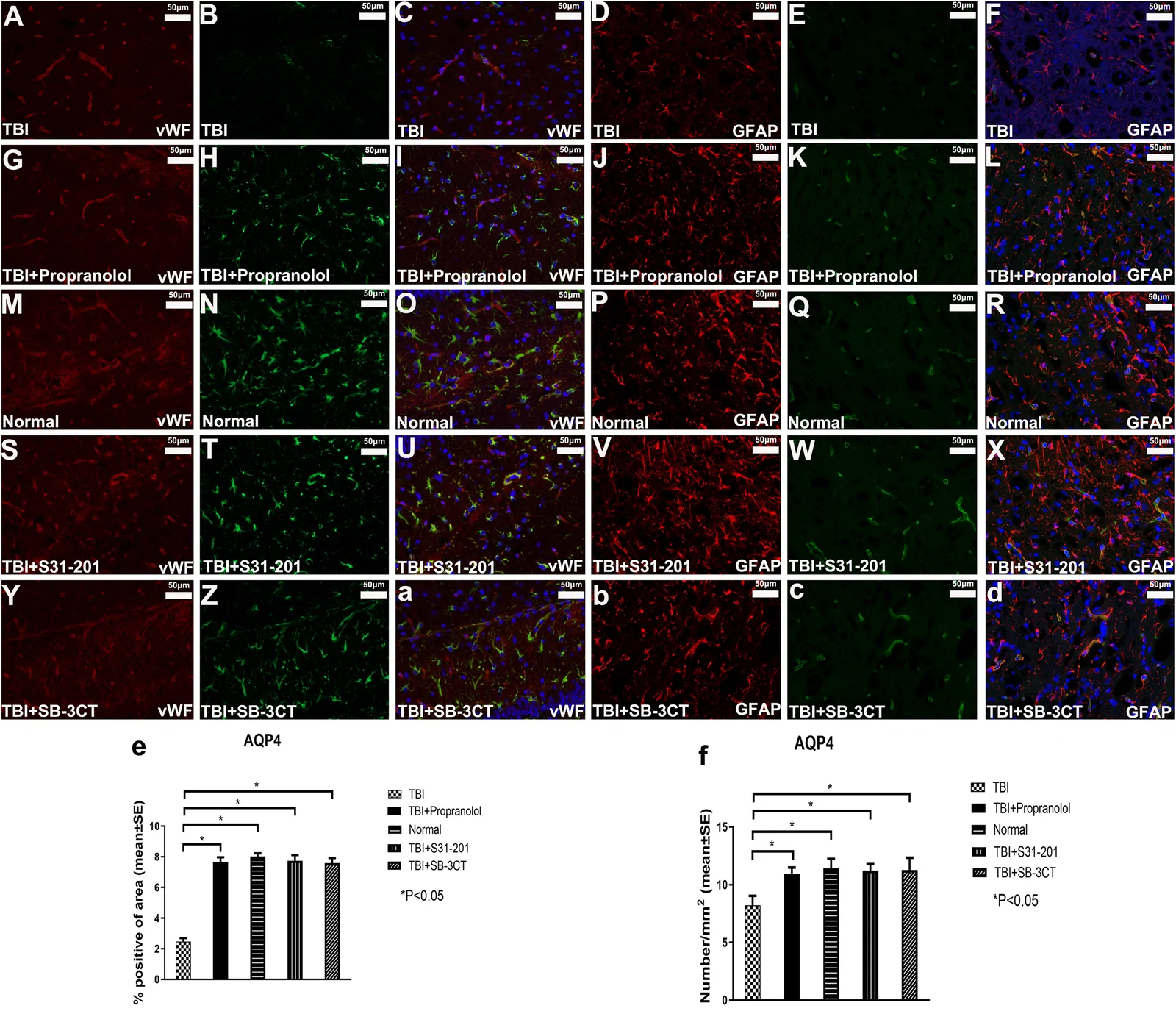
Perivascular and astrocytic AQP4 expression in rat brains. (A–C, G–I, M–O, S–U, Y–a, e) Propranolol, S31-201, and SB-3CT totally restored perivascular AQP4 (green) expression compared with untreated TBI rats. No statistically significant differences were detected among the three treatment groups and the normal group. Blood vessels were labeled with vWF (von Willebrand factor, red), and cell nuclei were counterstained with DAPI. (D–F, J–L, P–R, V–X, b–d, f) Propranolol, S31-201, and SB-3CT totally restored astrocytic AQP4 (green) expression compared with untreated TBI rats. No statistically significant differences were detected among the three treatment groups and the normal group. Astrocytes were labeled with glial fibrillary acidic protein (GFAP, red), and cell nuclei were counterstained with DAPI. (*p < 0.05, n = 6/group, 10 × 40 objective lens).

To elucidate the mechanism by which propranolol ameliorates TBI-induced impairment of glymphatic function, we quantified perivascular AQP4 expression and astrocyte levels in the brain. The results demonstrated that propranolol, S31-201, and SB-3CT totally restored perivascular and astrocytic AQP4 expression relative to untreated TBI rats. No statistically significant differences were identified among the three treatment groups and the normal group (Fig 4).

### Propranolol decreased p-STAT3/MMP2 expression after TBI

**Fig 5:**
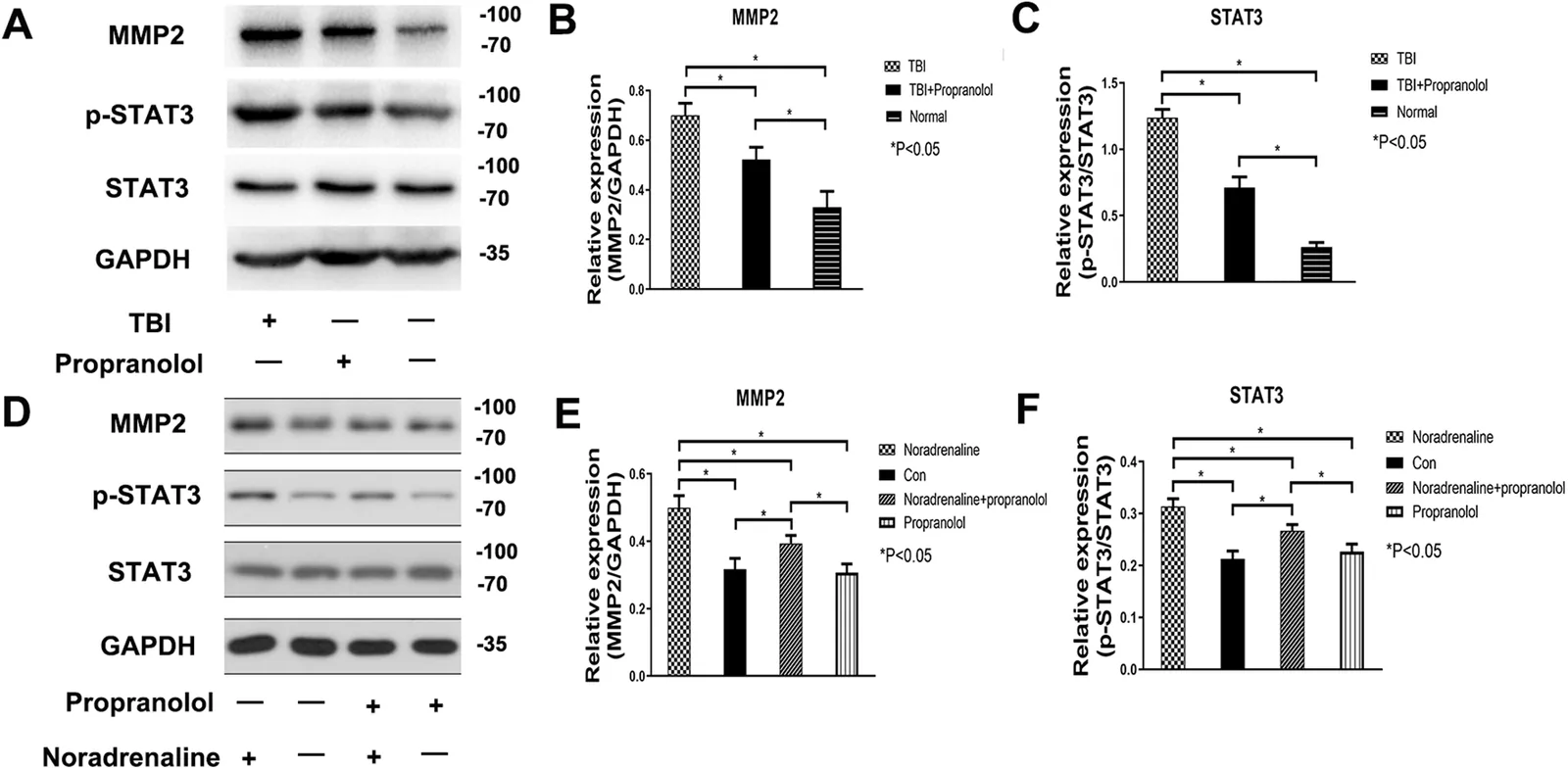
Expressions of p-STAT3/MMP2 in vivo and vitro. (A, B) MMP2 protein level was markedly decreased in propranolol-treated rats compared with TBI model rats. Nevertheless, propranolol could not fully restore MMP2 upregulation caused by TBI to the level of normal rats. GAPDH was applied as the loading reference. (A, C) Western blot quantification demonstrated that p-STAT3 expression was significantly lower in the propranolol intervention group than in the TBI group, whereas complete recovery of TBI-induced abnormal p-STAT3 expression was not achieved by propranolol treatment relative to normal rats. Total STAT3 was set as the loading control. (D, E) Incubation with noradrenaline led to significant upregulation of MMP2 in astrocytes. Additional propranolol treatment rescued the noradrenaline-mediated elevation in MMP2 expression, though full normalization to the control group level was not attained. No statistical difference was detected between the single propranolol group and the control group. GAPDH served as the loading control. (D, F) Noradrenaline stimulation significantly increased p-STAT3 levels in astrocytes, and this upregulation was partially counteracted by propranolol supplementation. However, p-STAT3 expression could not be fully restored to the baseline level of the control group. No statistical difference was observed between the single propranolol group and the control group. Total STAT3 was used as the loading control. (*p < 0.05).

To further explore the mechanism of propranolol-induced improvement of cognitive function and glymphatic system recovery after TBI, Western blot was used to test the expression of phosphorylated STAT3 (p-STAT3) and MMP2. The results showed that the levels of p-STAT3 and MMP2 were significantly downregulated in the propranolol-treated TBI rats, but propranolol could not completely reverse the increased levels of p-STAT3 and MMP2 after TBI compared with normal rats (Fig 5).

### Propranolol reversed the noradrenaline-mediated increased expression of p-STAT3/MMP2 in astrocytes

To further clarify the mechanism of propranolol-induced reduction in p-STAT3/MMP2 expression in TBI rats, we treated astrocytes with noradrenaline and propranolol. Western blot data showed that the levels of p-STAT3 and MMP2 were upregulated after astrocytes were incubated with noradrenaline. Subsequent addition of propranolol reversed the noradrenaline-induced elevation in p-STAT3 and MMP2 expression; however, propranolol failed to fully restore p-STAT3 and MMP2 levels to those of the control group. No significant difference was observed between the propranolol-only group and the control group (Fig 5).

### Propranolol antagonized the noradrenaline-mediated decline expression of AQP4 of astrocytes

**Fig 6:**
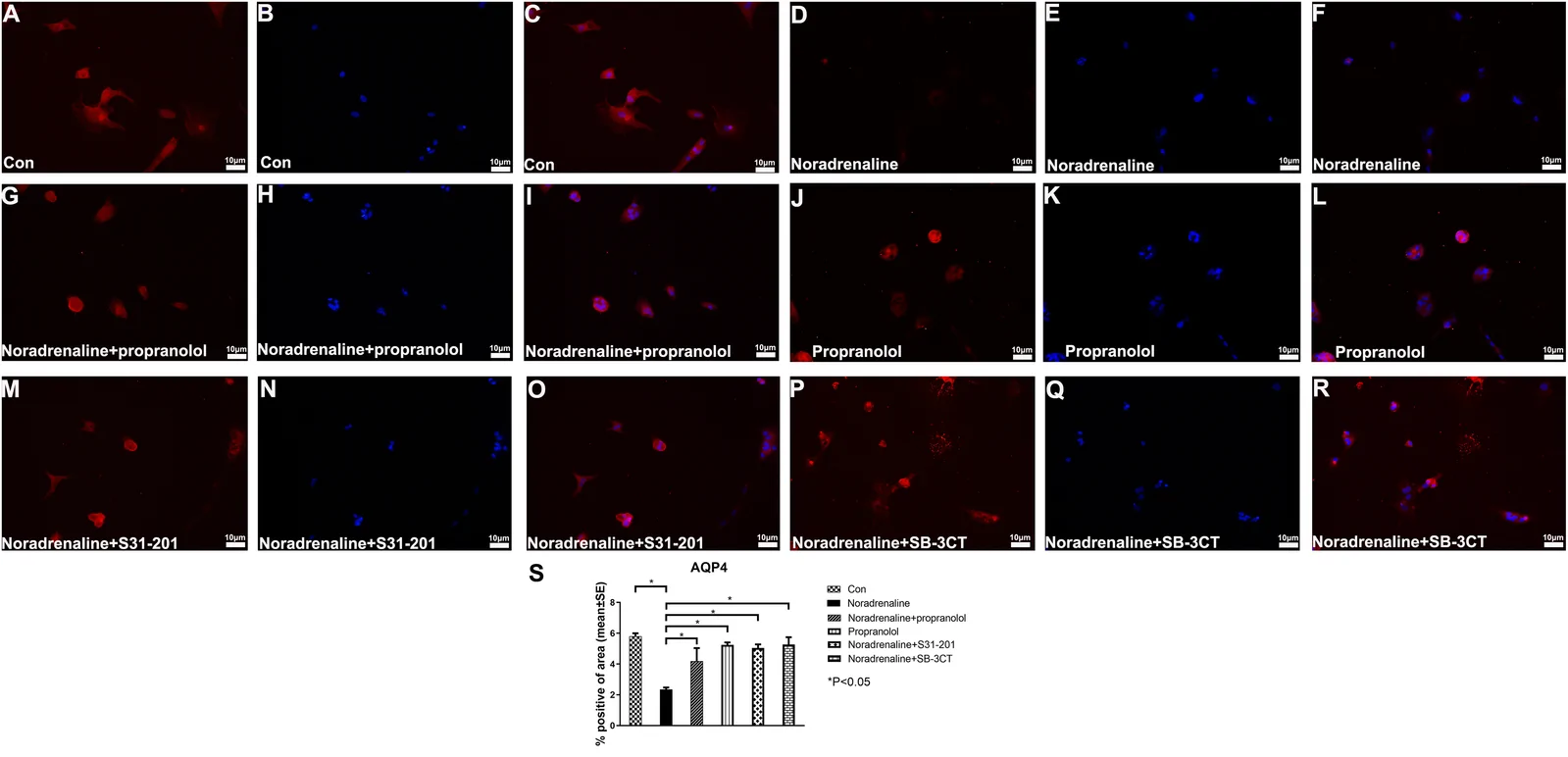
Expressions of astrocytic AQP4 in vitro. (D–F, S) AQP4 (red) expression was significantly decreased in astrocytes following noradrenaline treatment relative to the control group. (G–I, S) Treatment with propranolol fully restored the noradrenaline-evoked downregulation of AQP4 (red) in astrocytes. (J–L, S) No statistically significant difference in AQP4 (red) expression was detected between the propranolol single-treatment group and the control group. (M–O, S) S31-201 fully rescued the noradrenaline-induced reduction in astrocytic AQP4 (red) expression. (P–R, S) SB-3CT also completely reversed the noradrenaline-triggered decline in AQP4 (red) expression in astrocytes. Nuclei were counterstained with DAPI. *p < 0.05, 10 × 20 objective lens, 10 of fields and 6 of cells per field were counted).

To further elucidate how propranolol elevates AQP4 expression following TBI, astrocytes were subjected to treatments with noradrenaline alone or combined with propranolol, S31-201 or SB-3CT. Immunohistochemical staining was utilized to quantify AQP4 expression in astrocytes. Noradrenaline treatment markedly downregulated AQP4 levels in astrocytes, whereas co-administration of propranolol, S31-201 or SB-3CT fully rescued this noradrenaline-triggered AQP4 downregulation. (Fig 6)

## Discussion

TBI can trigger a range of symptoms including impaired cognitive function, headache, depression, fatigue, irritability and anxiety, which impair the quality of life in TBI patients [1]. Many patients with TBI obtain clinical benefits from β-blocker (BB) treatment. As a member of BBs, propranolol exerts superior therapeutic effects relative to other BBs owing to its lipophilic characteristics; it improves cerebral perfusion and reduces cerebral catabolism and oxygen consumption [24]. In the present study, we confirmed that propranolol is capable of ameliorating TBI-induced cognitive impairment. However, the exact mechanism underlying propranolol-mediated cognitive recovery remains unclear.

There are numbers of deposition of proteinaceous waste products (such as: p-tau and Aβ) in patients with chronic traumatic encephalopathy (CTE) and sleep disruption following TBI. These waste products are thought to drive neurodegenerative processes seen in Alzheimer’s disease (AD) and TBI [25]. The glymphatic system, responsible for metabolic waste clearance within the brain, has emerged as a promising therapeutic target for traumatic brain injury (TBI). Multiple pathological alterations following TBI compromise glymphatic function, including astrocyte dysfunction and impaired perivascular spaces (PVS) [26]. AQP4 is widely distributed in cerebral capillary endothelial cells, astrocytes and ependymocytes. Its expression is markedly downregulated at 6 hours after TBI. Cerebral contusion is frequently complicated by cerebral edema, which accounts for numerous clinical syndromes to TBI. Previous research has confirmed that TBI-induced brain edema is aggravated in AQP4 knockout rats, demonstrating that AQP4 is critical for regulating edema development post-TBI [27]. Meanwhile, the AQP4 expression in blood vessels and astrocytes plays an essential role in the glymphatic system [8]. Given the marked interindividual differences and diverse pathophysiological alterations following TBI, interventions aimed at restoring impaired glymphatic function could serve as a unified approach to resolve these interindividual and pathophysiological differences [28]. Therapeutic strategies targeting the glymphatic system for TBI have witnessed substantial progress over recent years. Restoring sleep is capable of mitigating TBI-related cognitive deficits. This is attributable to the fact that natural sleep and anesthesia both boost convective exchange between cerebrospinal fluid and interstitial fluid, thereby elevating the clearance efficiency of β-amyloid [29]. Fingolimod exerts effective therapeutic effects against TBI via re-establishing AQP4 polarization and enhancing glymphatic system [30]. Analogously, cannabidiol, exendin-4, minocycline and interleukin-33 can rescue TBI-induced glymphatic dysfunction either by modulating AQP4 polarization or elevating AQP4 expression level [31–34]. Multisensory gamma stimulation (40 Hz) rescues the amyloid-clearing function of the glymphatic system. This effect is achieved through re-establishing AQP4 polarization, expanding meningeal lymphatic vessels (mLVs), and orchestrating diverse cellular components to maintain proper glymphatic system [35]. Previous studies have suggested that adrenergic inhibitors enhance fluid outflow from brain parenchyma, ameliorate sleep disorders, and mitigate cerebral edema [36,37]. Accordingly, adrenergic inhibitors (such as propranolol) represented tight correlations with glymphatic function. Consistent with our unexpected experimental findings, propranolol attenuates cerebral p-tau and Aβ accumulation post-TBI via functional restoration of the glymphatic system. Further mechanistic validation verified that propranolol upregulates AQP4 expression in cerebrovascular endothelial cells and astrocytes, providing a partial molecular explanation for the recovered glymphatic activity triggered by propranolol. Collectively, our findings combined with earlier evidence identify AQP4 as a universal regulatory node governing glymphatic system for the majority of TBI therapeutic medications. Furthermore, additional clinical investigations have documented substantial auxiliary advantages of adrenergic blockade, including relief from sleep disturbances [37] and improvements in neuropsychiatric complications [36]. Accordingly, adrenergic inhibitors including propranolol confer more diverse therapeutic benefits than alternative TBI medications that solely target the glymphatic system alone. Nevertheless, translating adrenergic inhibition into clinical practice faces substantial obstacles, given that established clinical guidelines stress the necessity of stable cerebral perfusion for TBI patients. Further preclinical and clinical studies are therefore warranted to resolve this dilemma [38].

Propranolol is also implicated in the treatment of a wide range of other neurological illnesses. Accumulating evidence confirm that propranolol markedly attenuates tremors associated with Parkinson’s disease (PD) [39,40]. Its therapeutic outcome is likely regulated by beta‑1 adrenergic receptors located in the motor cortex [40]. Moreover, propranolol represents favorable therapeutic outcomes in Alzheimer’s disease (AD) and other cognitive impairments, as it strengthens cholinergic signaling via the suppression of butyrylcholinesterase activity [41]. On the contrary, existing research indicates that activating β2-adrenergic receptors can reverse memory deficits induced by amyloid-β. Propranolol exerts antagonistic effects and eliminates the beneficial pathological modifications mediated by β2-adrenergic receptor stimulation [42]. For this reason, the effect of propranolol across neurological disorders is still under debate. Further preclinical and clinical investigations are warranted to dissect the detailed molecular mechanisms of propranolol.

Accordingly, we further probed the molecular mechanism by which propranolol rescues AQP4 recovery after TBI. Dystroglycan (DG) is broadly distributed along the junction of the basement membrane and cell membrane across diverse tissues. DG is critical for sustaining proper AQP4 polarization in astrocytes. Previous studies have demonstrated that nonspecific matrix metalloproteinase (MMP) inhibitors normalize DG expression in astrocytes subjected to oxygen–glucose deprivation, confirming that MMP activity drives DG degradation in these cells. Although MMP-2 has been verified to cleave DG in cerebral ischemic injury, the precise regulatory pathway has not been fully clarified [10,43,44].

As a neurotransmitter and neuromodulator, norepinephrine (NE) participates widely in the physiological activities of neuronal and non-neuronal cells. Existing data reveal that NE elevates phosphorylated signal transducer and activator of transcription 3 (p-STAT3) level in pancreatic cancer cells in a dose-dependent fashion. This NE-mediated STAT3 phosphorylation can be effectively suppressed by propranolol (a β-adrenergic receptor inhibitor, β-ARI). In turn, pharmacological inhibition of STAT3 signaling results in significant downregulation of MMP2 [11]. Intriguingly, prior work has documented that the glymphatic system exhibits a markedly higher clearance efficiency during sleep compared with wakefulness [45]. The central NE–locus coeruleus (LC) noradrenergic system is quiescent in the sleeping brain, thereby enabling accelerated removal of cerebral metabolic waste [25].

We therefore hypothesized that the NE/β-ARI /STAT3/MMP2 signaling cascade mediates AQP4 depletion after traumatic brain injury. Our in vivo results showed that propranolol markedly suppressed the upregulation of p-STAT3 and MMP2 in TBI animals, offering a partial mechanistic explanation for propranolol-dependent AQP4 recovery. Consistent with these in vivo observations, in vitro assays confirmed that propranolol abolished norepinephrine-triggered elevation of p-STAT3/MMP2 and rescued reduced AQP4 level in astrocytes, solidifying our initial hypothesis. Like propranolol, the STAT3 inhibitor S31-201 and MMP2 inhibitor SB-3CT mitigated TBI-associated cognitive dysfunction and restored defective glymphatic system. In TBI rat models, administration of either agent reduced p-tau and Aβ accumulation and maintained white matter integrity. S31-201 and SB-3CT each elevated AQP4 expression in cerebral blood vessels and astrocytes and counteracted norepinephrine-induced AQP4 repression in astrocytes. Taken together, these data demonstrate that propranolol ameliorates cognitive impairment and facilitates glymphatic functional restoration post-TBI by targeting the STAT3/MMP2 signaling pathway.

This study provides a new mechanistic insight and a promising therapeutic candidate (propranolol) for cognitive rehabilitation after TBI. Nevertheless, several limitations of this work should be acknowledged. While we confirmed the cognitive-protective efficacy of propranolol in TBI models, we could not elucidate why this drug failed to fully normalize memory function, myelin density and aberrant p-STAT3/MMP2 expression compared with normal rats. In addition, our unpublished data suggest that high-dose propranolol (10 mg/kg) impairs cognitive recovery post-TBI. This raises the possibility that uncharacterized pathways mediate both the favorable and adverse outcomes of propranolol treatment. Previous literature has reported that propranolol modulates neuroinflammation, a biological process that was not examined in our current study. Numerous brain cell populations express distinct α- and β-adrenergic receptor subtypes, and their individual roles in TBI pathogenesis remain poorly defined. Moreover, direct regulatory effects of propranolol on AQP4 polarization have not yet been verified and require further experimental validation [7].

## Conclusion

Propranolol may improve cognitive function and glymphatic system recovery after TBI through STAT3/MMP2 pathway.

## Supporting information

S1 File Western bolt. (PDF)
