# Supplementary material for: Propranolol improves cognitive function and glymphatic system recovery through STAT3/MMP2 pathway after TBI
Source: PLoS One. 2026 Aug 21;21(8):e0353852. doi: 10.1371/journal.pone.0353852 (PMC13496413; doi:10.1371/journal.pone.0353852)
Supplement: Supplementary file 1 [file pone.0353852.s001.pdf]

- **Western blot：**
- Briefly, equal volumes of protein samples were separated by SDS-PAGE and transferred onto polyvinylidene difluoride (PVDF) membranes (Millipore, Billerica, MA, USA). After blocking, the membranes were incubated overnight at 4 °C with primary antibodies against MMP2 (1:2000, Abcam, Cambridgeshire, UK), STAT3 (1:1000, Abcam, Cambridgeshire, UK), phosphorylated STAT3 (p-STAT3, 1:5000, Abcam, Cambridgeshire, UK), and GAPDH (1:5000, Solarbio, Beijing, China; 1:2000, Abcam, Cambridgeshire, UK), which served as an internal reference. Subsequently, the membranes were incubated with horseradish peroxidase (HRP)-linked secondary antibodies (1:1500; Santa Cruz Biotechnology, USA) for 2 h at room temperature. Protein bands were visualized using SuperSignal West Pico Chemiluminescent Substrate (Pierce, Rockford, IL, USA). Band intensity was measured with Image J software (NIH), and the relative expression levels were normalized to GAPDH.

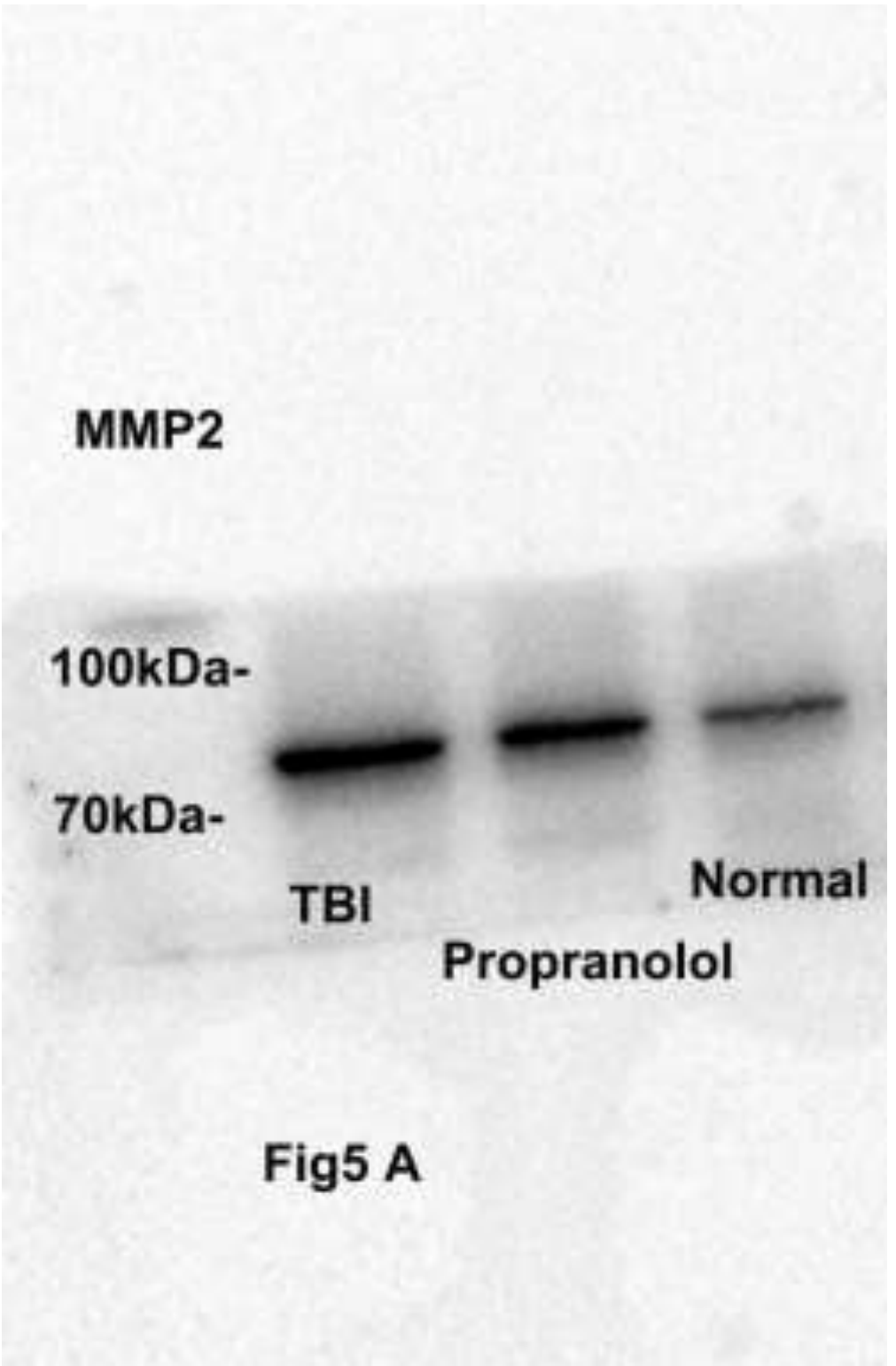
MMP2
100kDa-
70kDa-
TBI
Propranolol
Normal
Fig5 A

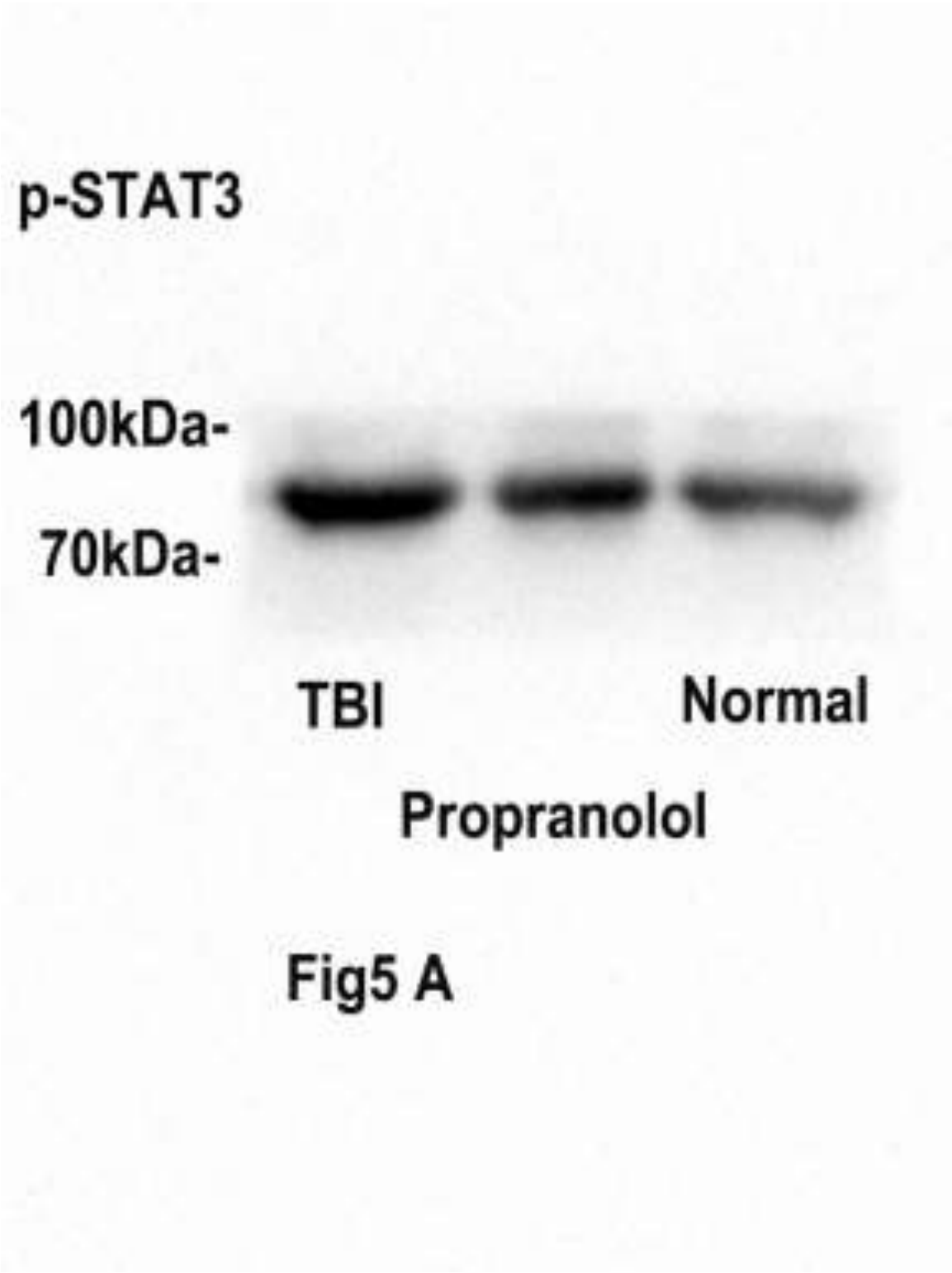
p-STAT3
100kDa-
70kDa-
TBI
Propranolol
Normal
Fig5 A

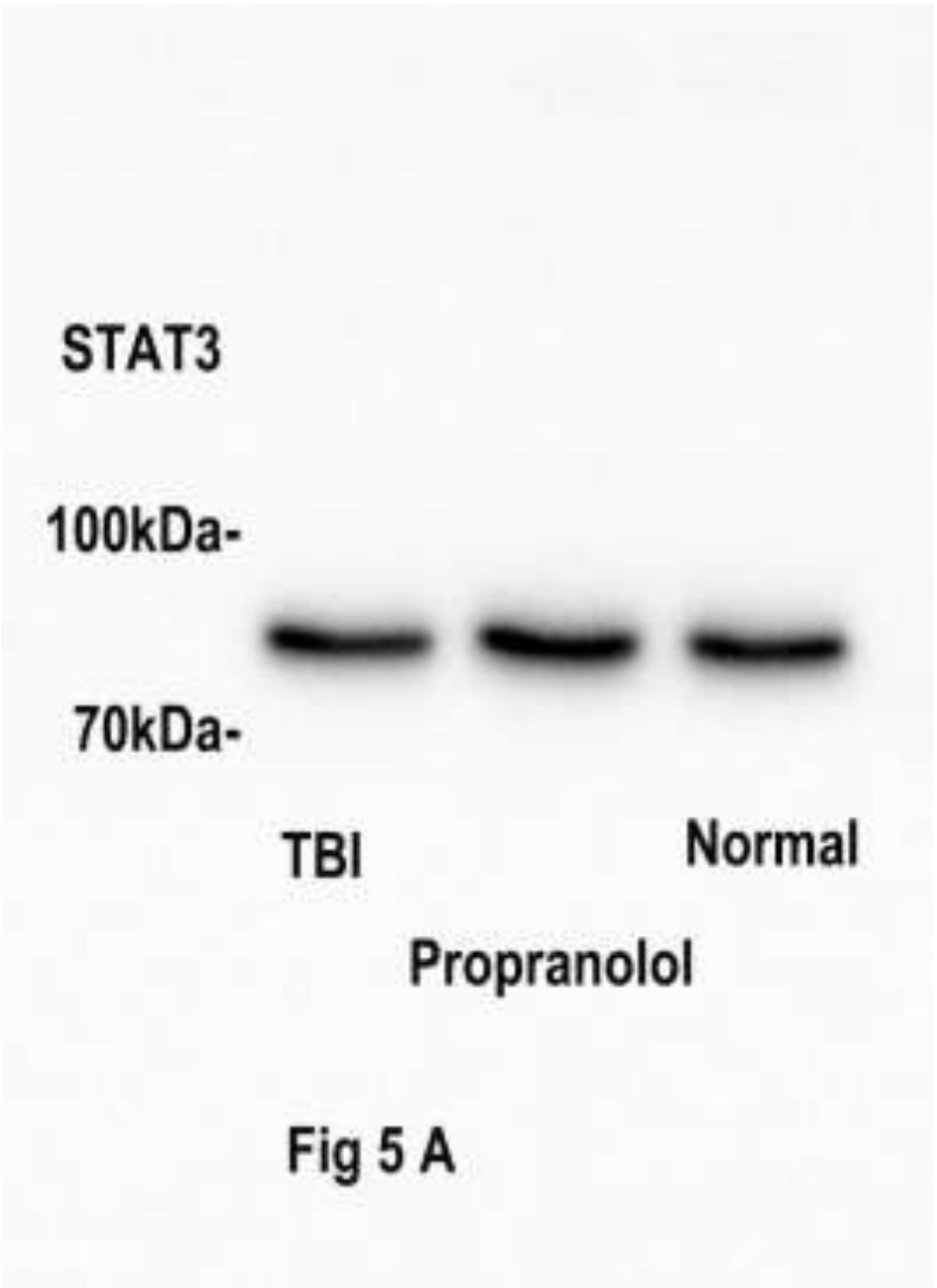
STAT3
100kDa-
70kDa-
TBI
Propranolol
Normal
Fig 5 A

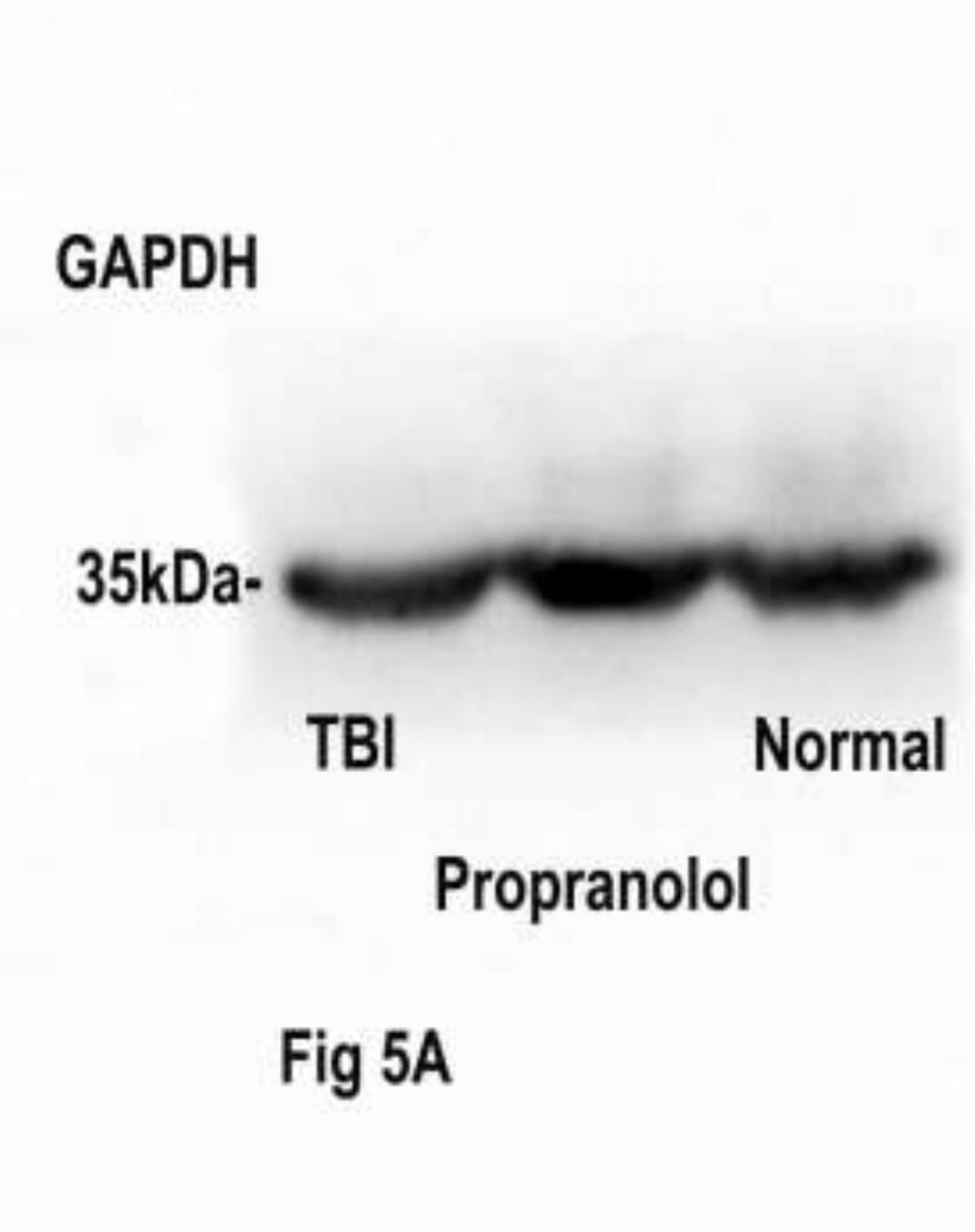
GAPDH
35kDa-
TBI
Propranolol
Normal
Fig 5A

Fig5 D

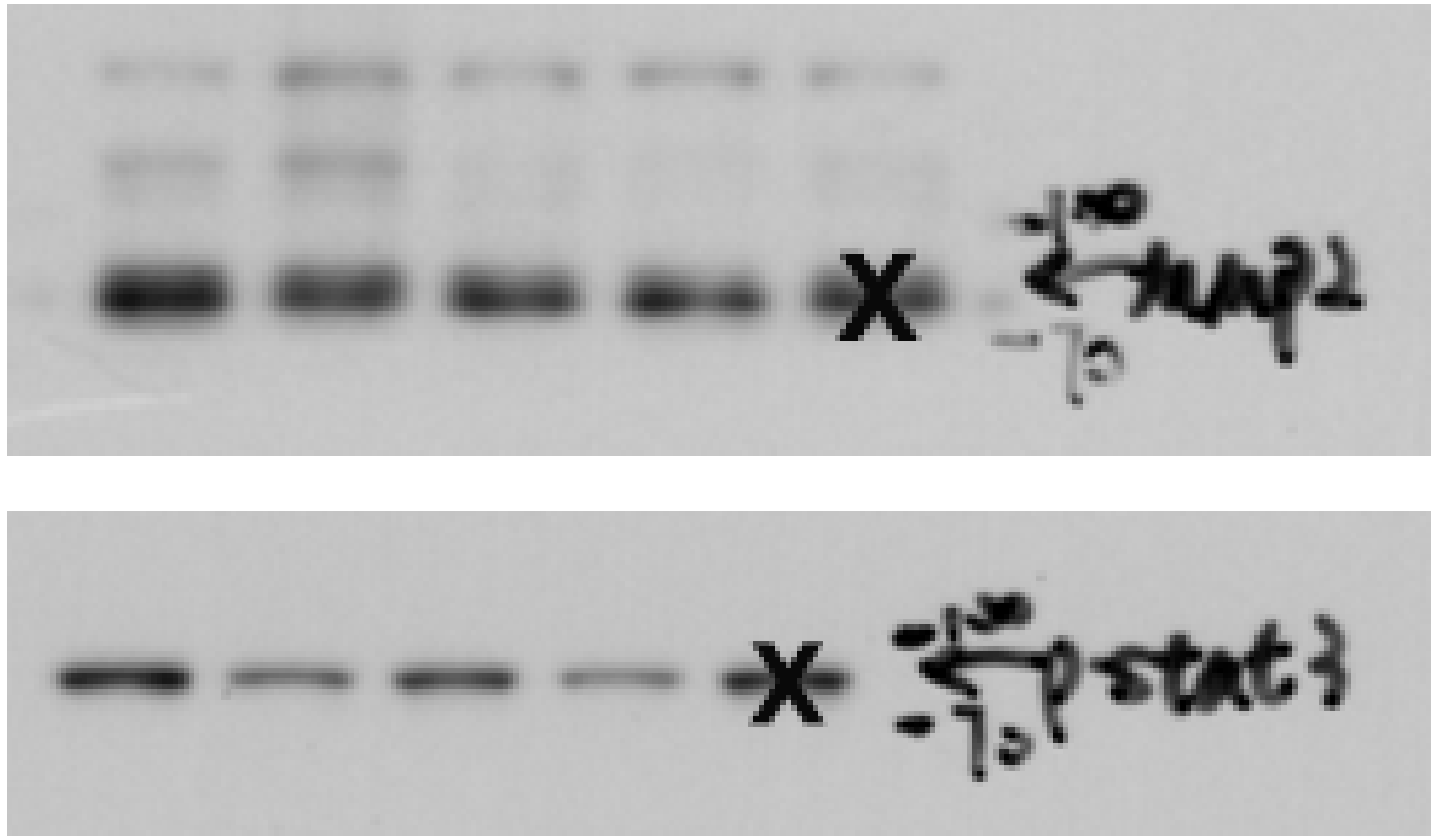
Noradrenaline
Control
Propranolol+Noradrenaline
Propranolol

Fig5 D

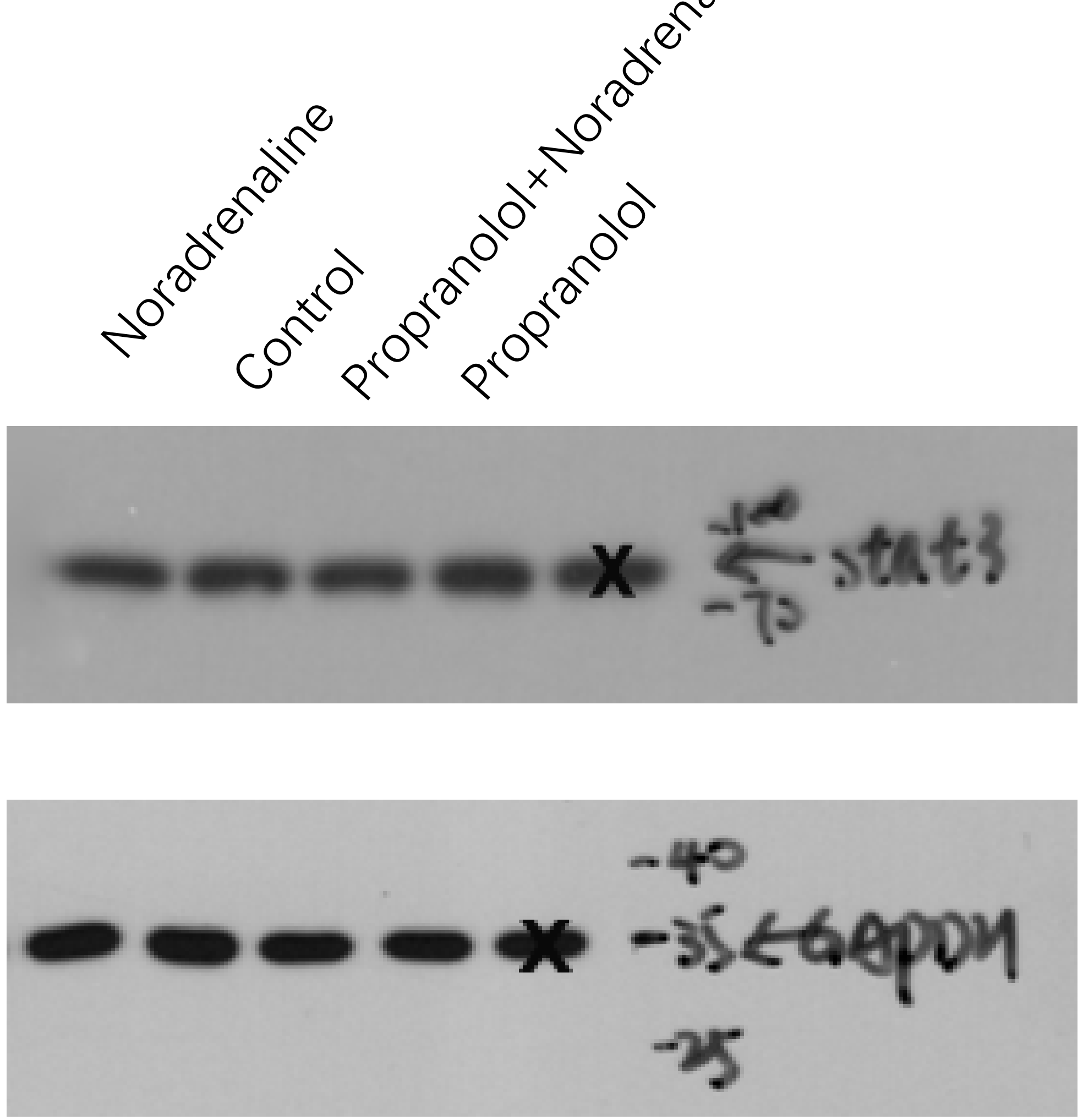

Noradrenaline
Control
Propranolol+Noradrenaline
Propranolol
stat3
-40
-35
GAPDH
-25
