# Supplementary material for: Propranolol improves cognitive function and glymphatic system recovery through STAT3/MMP2 pathway after TBI
Source: PLoS One. 2026 Aug 21;21(8):e0353852. doi: 10.1371/journal.pone.0353852 (PMC13496413; doi:10.1371/journal.pone.0353852)
Supplement: Supplementary file 2 [file pone.0353852.s003.docx]

Dear Reviewer:

Thank you for your valuable comments which are very helpful for revising and improving our paper. We have carefully studied your comments and made corresponding revision. Revised portion are marked in red in the submitted paper. The main corrections in the paper and the responds to the reviewer’s comments are as follows:

Reviewer’s comments:

1. Animal Experiment Design
Group Completeness: The study only sets up the TBI group and the TBI + propranolol group, lacking a normal control group (healthy rats that did not undergo TBI surgery). The absence of the normal control group makes it impossible to clearly determine the effect of propranolol on the cognitive function, lymphoid system, and related protein expression of normal rats, and also makes it difficult to completely distinguish the pathological damage of TBI itself from the specific effect of propranolol intervention. It is recommended to supplement the normal control group to more comprehensively evaluate the effect of propranolol.

**Reply:** Thanks to you for your excellent comment. We added normal control group (healthy rats that did not undergo TBI surgery) in our study. It turned out that even though propranolol could improve cognitive function after TBI, propranolol could not completely reverse the short-term memory, long-term memory and spatial learning deficits after TBI as compared to normal rats. On the other hand, propranolol could totally reverse TBI-induced p-tau/Aβ deposition. Myelin densities were significantly recovered in the brains of propranolol-treated rats, but propranolol could not completely reverse the myelin densities deficits after TBI as compared to normal rats. Also, propranolol could completely reverse the glymphatic dysfunction after TBI. Moreover, propranolol totally recovered the AQP4 accumulation around astrocyte and vessels after TBI as compared to normal group. The results showed that the level of p-STAT3/MMP2 were significantly downregulated in the propranolol-treated rats, but propranolol could not completely reverse the increased level of p-STAT3/MMP2 after TBI as compared to normal rats. In vitro, it turned out that propranolol reversed the noradrenaline-mediated increased expression of p-STAT3/MMP2 in astrocytes, but propranolol could not totally restore the noradrenaline-mediated expression of p-STAT3/MMP2 as compared to control group. We found that noradrenaline could decrease the lever of AQP4 expression of astrocytes. On the other hand, it showed that propranolol totally reversed the noradrenaline-mediated decline expression of AQP4 of astrocytes.

2. Detection Method Details

Lymphoid System Function Detection: The method of injecting 70kD dextran into the cerebellopontine cistern and detecting fluorescence density is used to evaluate the lymphoid system function. However, the accuracy control measures for the injection site (such as whether it is precisely located by a stereotactic instrument and whether there is a record of the injection position after the injection for anatomical verification) are not explained. Deviation in the injection site may lead to uneven distribution of dextran and affect the reliability of the quantitative results of fluorescence density. It is recommended to supplement the experimental details of precise control of the injection site.

**Reply:** Thanks to you for your excellent comment. The detail of Lymphoid System Function Detection was added as follow: Anesthetized rats were fixed in a stereotaxic frame and a 30GA needle was inserted into the central cisterna magna to avoid uneven distribution of dextran. 5µl of Dextran (Sigma, MW: 70 kD, USA) was injected into the cisterna magna of TBI rats over 5 minutes using a syringe pump at a flow rate of 2μl/min. Rats were sacrificed at 30 minutes from the start infusion. The rats were transcardially perfused with 0.9% saline followed by 4% paraformaldehyde. 80 mm thick sections were cut and imaged using a 10x objective of a laser-scanning confocal microscope (Zeiss LSM 510 NLO, Carl Zeiss, Germany). MCID image analysis system (Imaging Research, St. Catharines, Ontario, Canada) was used to quantify the fluorescence density. The data were presented as percentage of positive fluorescence area in the brain cross section.

3. Discussion and Conclusion

The discussion only compares the differences between propranolol and other β receptor blockers (such as lipophilicity), but does not fully combine the recent research progress on the regulation of the lymphoid system after TBI (such as the effects of other drugs and physical intervention methods on the lymphoid system), nor discusses the similarities and differences between the results of this study and other studies on the role of propranolol in neurological diseases (such as the research conclusions of propranolol in Alzheimer's disease and Parkinson's disease). It is recommended to expand the discussion scope, place this study in a broader research context, and highlight the innovation and limitations of the study.,This manuscript innovatively proposes a hypothesis that propranolol may regulate the function of the lymphoid system through the STAT3/MMP2 pathway to improve cognition, and this hypothesis is verified through in vitro and in vivo experiments. The research design is logically coherent, and the technical route is clear. It provides a new mechanism explanation and potential therapeutic direction for the repair of cognitive function after TBI, and has certain scientific value and clinical reference significance. However, the research still has areas that can be improved in terms of experimental design details, mechanism depth, and result interpretation. Further supplementation and optimization are required.

**Reply:** Thanks to you for your good comment. As reviewer suggested, we expanded the discussion scope. The changed discussion section was labeled with red in the main manuscript.

**Discussion:**

TBI could cause a series of symptoms, such as declined cognitive function, headaches, depression, fatigue, irritability, and anxiety, which influences living quality of TBI patients [1]. Multiple TBI patients benefit from administration of β-blockers (BBs). As one of BBs, propranolol may have more benefits than other BBs because of the lipophilic properties, increasing cerebral perfusion and decreasing catabolism and oxygen consumption of brain [20]. In this research, we conformed that propranolol could enhance defective cognitive function after TBI. But, the mechanism of propranolol-induced cognitive function recovery is still unknown.

These are numbers of proteinaceous waste products (for example: p-tau and Aβ etc.) deposition in chronic traumatic encephalopathy (CTE) and sleep disruption patients after TBI. The waste products are thought to contribute to neurodegeneration processes as Alzheimer’s disease (AD) and TBI [21]. The glymphatic system which helps to clear the waste products in brain is a promising treatment target after TBI. There are lots of factors that could influence the function of glymphatic system, like astrocyte dysfunction and perivascular spaces (PVS) disruption after TBI [22]. AQP4 is wildly distributed in capillary endothelial cells, astrocytes and ependymocytes in the brain. The level of AQP4 is significantly downregulated at 6h after TBI. The cerebral contusion is usually accompanied with cerebral edema, which takes responsible for a variety of syndromes after TBI. It has showed that the TBI-induced edema is deteriorated in the AQP4 knock-out rat which suggested that AQP4 plays an essential role in edema after TBI [23]. Meanwhile, the expression of AQP4 of vessels and astrocytes play an essential role in glymphatic system [8]. Thus, due to the ubiquitous heterogeneity of individuals, conditions, and pathophysiological mechanisms after TBI [24], treating glymphatic system dysfunction maybe a common pathway underlying these variations. The potential TBI treatment targeted at glymphatic system has made big strides these years. Sleep recovery may relieve TBI-induced cognitive impairment. Because, natural sleep or anesthesia could increase convective exchange of cerebrospinal fluid with interstitial fluid which may help to increase the rate of β-amyloid clearance[25]. Administering fingolimod could treat TBI effectively by promoting AQP4 repolarization and improving glymphatic system function [26]. Similar to fingolimod, cannabidiol, exendin-4, minocycline and interleukin-33 could all recover the function of TBI through regulating AQP4 polarization or increasing the AQP4 expression [27-30]. Multisensory gamma stimulation (40 Hz stimulation) can restore glymphatic system to clear amyloid by enhancing the polarization of AQP4, increasing the volume of meningeal lymphatic vessels (mLVs) and synchronizing multiple cells in glymphatic flow [31]. Previous studies suggest that adrenergic inhibitors promote fluid drainage from brain tissue, alleviate sleep disturbance, and decrease brain edema [32, 33]. Thus, it seems that adrenergic inhibitors, like propranolol, have a strong relationship with glymphatic system. In this study, we unexpectedly found that propranolol decreased p-tau and Aβ deposition in brain by glymphatic system recovery after TBI. The results also revealed the facts that propranolol could restore the expression of AQP4 on cerebral blood vessels and astrocytes, which may partly explain the mechanism of propranolol-induced glymphatic system recovery. To sum up, Our and previous studies suggest that AQP4 is a common regulated target that influencing the glymphatic system for the most of treating TBI drugs. Moreover, Other clinical studies have found other considerable beneficial effects of adrenergic inhibition, such as alleviating sleep disturbance [33], improving psychiatric disorders [32]. Thus, adrenergic inhibition, like propranolol, may reveal more diversified beneficial effects than other treating TBI drugs that targeted glymphatic system only. But, the clinical translation of adrenergic inhibition has been challenging, because maintaining cerebral blood perfusion is critical for the TBI patients according to existing guidelines [34]. Thus, this conflict requires further basic and clinical research to resolve.

Propranolol also has significant relationship with other neurological diseases. These are studies show that propranolol could reduce Parkinson’s disease (PD) tremor effectively [35, 36]. The effect of propranolol is may influenced by beta-1 adrenergic receptor in the motor cortex.[36] Also, propranolol represents promising therapeutic effects in Alzheimer's disease (AD) and other cognitive disorders through cholinergic enhancement via butyrylcholinesterase inhibition.[37] In the contrary, there is study shown that the activation of beta-2 adrenergic receptor ameliorated amyloid-β-induced memory deficits. Inhibition of beta-2 adrenergic receptor by propranolol blocks the activation of beta-2 adrenergic receptor-induced improvements in pathological changes [38]. Thus, what role of propranolol is played in the neurological diseases is still controversial. More basic and clinical researches are also needed to explore the mechanism of propranolol for the future.

Hence, we further explored what mechanism induced propranolol-mediated AQP4 recovery of TBI. Dystroglycan (DG) widely expresses at the interface between the basement membrane and the cell membrane in various different kinds of tissues. DG plays an essential role in for maintaining the polarization of AQP4 in astrocytes. There are evidences indicate that nonspecific matrix metalloproteinase (MMP) inhibitors reverse the level of DG in astrocyte under oxygen–glucose deprivation condition, which implies that MMP takes responsible for the astrocyte-induced DG reduction. There are evidences show that MMP-2 is involved in cleavage of DG in cerebral ischemia, but the mechanism remains unclear [10, 39, 40]. Norepinephrine (NE), as a neurotransmitter and neuromodulator, plays a major role in multiple activities of neuronal or non-neuronal cells. The evidences show that NE promotes the expression of phosphorylated signal transducer and activator of transcription 3 (p-STAT3) dependent on a concentration-dependent manner in pancreatic cancer cells. NE-induced phosphorylation of STAT3 is inhibited by propranolol (aβ-adrenergic receptor inhibitor, β-ARI). Also, the level of MMP2 is downregulated through blocking STAT3 [11]. Interestingly, it is reported that clearance rate of glymphatic system is faster during sleep than during waking hours [41]. In the brain, the NE-the locus coeruleus (LC) system is shut down during sleep which may help to potentate more rapid clearance of waste products [21]. Thus, we speculated that NE/β-ARI/STAT3 signaling pathway may take responsible for deficiency of AQP4 after TBI. In the present study, we found that propranolol decreased p-STAT3/MMP2 expression after TBI in vivo which may partly explain the mechanism of propranolol-mediated AQP4 recovery of TBI. The vitro research showed that propranolol reversed the noradrenaline-mediated increased expression of p-STAT3/MMP2 and decline expression of AQP4 of astrocytes, which further confirmed our hypothesis.

Our study provides a new mechanism explanation and potential therapeutic direction of propranolol for the repair of cognitive function after TBI. But there are still lots of limitations in our study. Even though, we confirmed that propranolol improved cognitive function after TBI. But we could not explain why propranolol could not completely reverse the memory deficits, myelin densities deficits and expression of p-STAT3/MMP2 after TBI as compared to normal rats. Moreover, in our study (data not seen), high dose of propranolol (10mg/kg) may harm the cognitive function after TBI. Thus, we hypothesize if there are other mechanisms of propranolol-mediated benefits or disadvantages. As shown in other study, propranolol played an important role in regulating inflammation which was not seen in our study. There are different adrenergic receptors (α and β) in multiple types of cells in the brain. What roles of differential adrenergic receptors are played in TBI is still need further explored. Whether propranolol could influence AQP4 polarization directly still need to be further explored [7].

Thank you for your constructive comments. We tried our best to improve the manuscript and made some changes in the manuscript. These changes will not influence the content and framework of the paper. We appreciate for Reviewer’ warm work earnestly, and hope that the correction will meet with approval.

Once again, thank you very much for your comments and suggestions.

**References：**

1.McInnes K, Friesen CL, MacKenzie DE, Westwood DA, Boe SG. Correction: Mild Traumatic Brain Injury (mTBI) and chronic cognitive impairment: A scoping review. PloS one. 2019;14(6):e0218423. doi: 10.1371/journal.pone.0218423. PubMed PMID: 31185044; PubMed Central PMCID: PMC6559670.

2.Ramos-Cejudo J, Wisniewski T, Marmar C, Zetterberg H, Blennow K, de Leon MJ, et al. Traumatic Brain Injury and Alzheimer's Disease: The Cerebrovascular Link. EBioMedicine. 2018;28:21-30. doi: 10.1016/j.ebiom.2018.01.021. PubMed PMID: 29396300; PubMed Central PMCID: PMC5835563.

3.Iliff JJ, Chen MJ, Plog BA, Zeppenfeld DM, Soltero M, Yang L, et al. Impairment of glymphatic pathway function promotes tau pathology after traumatic brain injury. The Journal of neuroscience : the official journal of the Society for Neuroscience. 2014;34(49):16180-93. doi: 10.1523/JNEUROSCI.3020-14.2014. PubMed PMID: 25471560; PubMed Central PMCID: PMC4252540.

4.Khalili H, Ahl R, Paydar S, Sjolin G, Cao Y, Abdolrahimzadeh Fard H, et al. Beta-Blocker Therapy in Severe Traumatic Brain Injury: A Prospective Randomized Controlled Trial. World journal of surgery. 2020;44(6):1844-53. doi: 10.1007/s00268-020-05391-8. PubMed PMID: 32002583.

5.Solito E, Sastre M. Microglia function in Alzheimer's disease. Frontiers in pharmacology. 2012;3:14. doi: 10.3389/fphar.2012.00014. PubMed PMID: 22363284; PubMed Central PMCID: PMC3277080.

6.Acosta C, Anderson HD, Anderson CM. Astrocyte dysfunction in Alzheimer disease. Journal of neuroscience research. 2017;95(12):2430-47. doi: 10.1002/jnr.24075. PubMed PMID: 28467650.

7.O'Donnell J, Zeppenfeld D, McConnell E, Pena S, Nedergaard M. Norepinephrine: a neuromodulator that boosts the function of multiple cell types to optimize CNS performance. Neurochemical research. 2012;37(11):2496-512. doi: 10.1007/s11064-012-0818-x. PubMed PMID: 22717696; PubMed Central PMCID: PMC3548657.

8.Xia M, Yang L, Sun G, Qi S, Li B. Mechanism of depression as a risk factor in the development of Alzheimer's disease: the function of AQP4 and the glymphatic system. Psychopharmacology. 2017;234(3):365-79. doi: 10.1007/s00213-016-4473-9. PubMed PMID: 27837334.

9.Iliff JJ, Wang M, Liao Y, Plogg BA, Peng W, Gundersen GA, et al. A paravascular pathway facilitates CSF flow through the brain parenchyma and the clearance of interstitial solutes, including amyloid beta. Science translational medicine. 2012;4(147):147ra11. doi: 10.1126/scitranslmed.3003748. PubMed PMID: 22896675; PubMed Central PMCID: PMC3551275.

10.Yan W, Zhao X, Chen H, Zhong D, Jin J, Qin Q, et al. beta-Dystroglycan cleavage by matrix metalloproteinase-2/-9 disturbs aquaporin-4 polarization and influences brain edema in acute cerebral ischemia. Neuroscience. 2016;326:141-57. doi: 10.1016/j.neuroscience.2016.03.055. PubMed PMID: 27038751.

11.Guo K, Ma Q, Li J, Wang Z, Shan T, Li W, et al. Interaction of the sympathetic nerve with pancreatic cancer cells promotes perineural invasion through the activation of STAT3 signaling. Molecular cancer therapeutics. 2013;12(3):264-73. doi: 10.1158/1535-7163.MCT-12-0809. PubMed PMID: 23288783.

12.Ley EJ, Park R, Dagliyan G, Palestrant D, Miller CM, Conti PS, et al. In vivo effect of propranolol dose and timing on cerebral perfusion after traumatic brain injury. The Journal of trauma. 2010;68(2):353-6. doi: 10.1097/TA.0b013e3181c8269a. PubMed PMID: 20154548.

13.Niu F, Qian K, Qi H, Zhao Y, Jiang Y, Jia W, et al. CPCGI Reduces Gray and White Matter Injury by Upregulating Nrf2 Signaling and Suppressing Calpain Overactivation in a Rat Model of Controlled Cortical Impact. Neuropsychiatric disease and treatment. 2020;16:1929-41. doi: 10.2147/NDT.S266136. PubMed PMID: 32904488; PubMed Central PMCID: PMC7455756.

14.Lu H, Jiang R, Tao X, Duan C, Huang J, Huan W, et al. Expression of Dixdc1 and its Role in Astrocyte Proliferation after Traumatic Brain Injury. Cellular and molecular neurobiology. 2017;37(6):1131-9. doi: 10.1007/s10571-016-0446-0. PubMed PMID: 27873129.

15.Lee RK, Araki W, Wurtman RJ. Stimulation of amyloid precursor protein synthesis by adrenergic receptors coupled to cAMP formation. Proceedings of the National Academy of Sciences of the United STATes of America. 1997;94(10):5422-6. doi: 10.1073/pnas.94.10.5422. PubMed PMID: 9144253; PubMed Central PMCID: PMC24694.

16.Yu P, Venkat P, Chopp M, Zacharek A, Shen Y, Liang L, et al. Deficiency of tPA Exacerbates White Matter Damage, Neuroinflammation, Glymphatic Dysfunction and Cognitive Dysfunction in Aging Mice. Aging and disease. 2019;10(4):770-83. doi: 10.14336/AD.2018.0816. PubMed PMID: 31440383; PubMed Central PMCID: PMC6675536.

17.Xie BS, Wang YQ, Lin Y, Mao Q, Feng JF, Gao GY, et al. Inhibition of ferroptosis attenuates tissue damage and improves long-term outcomes after traumatic brain injury in mice. CNS neuroscience & therapeutics. 2019;25(4):465-75. doi: 10.1111/cns.13069. PubMed PMID: 30264934; PubMed Central PMCID: PMC6488926.

18.Smith AJ, Akdemir G, Wadhwa M, Song D, Verkman AS. Application of fluorescent dextrans to the brain surface under constant pressure reveals AQP4-independent solute uptake. The Journal of general physiology. 2021;153(8). doi: 10.1085/jgp.202112898. PubMed PMID: 34128962; PubMed Central PMCID: PMC8210582.

19.Yu P, Li S, Zhang Z, Wen X, Quan W, Tian Q, et al. Progesterone-mediated angiogenic activity of endothelial progenitor cell and angiogenesis in traumatic brain injury rats were antagonized by progesterone receptor antagonist. Cell proliferation. 2017;50(5). doi: 10.1111/cpr.12362. PubMed PMID: 28752929; PubMed Central PMCID: PMC6529122.

20.Schroeppel TJ, Sharpe JP, Magnotti LJ, Weinberg JA, Clement LP, Croce MA, et al. Traumatic brain injury and beta-blockers: not all drugs are created equal. The journal of trauma and acute care surgery. 2014;76(2):504-9; discussion 9. doi: 10.1097/TA.0000000000000104. PubMed PMID: 24458058.

21.Sullan MJ, Asken BM, Jaffee MS, DeKosky ST, Bauer RM. Glymphatic system disruption as a mediator of brain trauma and chronic traumatic encephalopathy. Neuroscience and biobehavioral reviews. 2018;84:316-24. doi: 10.1016/j.neubiorev.2017.08.016. PubMed PMID: 28859995.

22.Peng S, Liu J, Liang C, Yang L, Wang G. Aquaporin-4 in glymphatic system, and its implication for central nervous system disorders. Neurobiol Dis. 2023;179:106035. Epub 2023/02/17. doi: 10.1016/j.nbd.2023.106035. PubMed PMID: 36796590.

23.Zhang C, Chen J, Lu H. Expression of aquaporin-4 and pathological characteristics of brain injury in a rat model of traumatic brain injury. Molecular medicine reports. 2015;12(5):7351-7. doi: 10.3892/mmr.2015.4372. PubMed PMID: 26459070; PubMed Central PMCID: PMC4626127.

24.Covington NV, Duff MC. Heterogeneity Is a Hallmark of Traumatic Brain Injury, Not a Limitation: A New Perspective on Study Design in Rehabilitation Research. Am J Speech Lang Pathol. 2021;30(2S):974-85. Epub 2021/02/09. doi: 10.1044/2020_AJSLP-20-00081. PubMed PMID: 33556261.

25.Xie L, Kang H, Xu Q, Chen MJ, Liao Y, Thiyagarajan M, et al. Sleep drives metabolite clearance from the adult brain. Science. 2013;342(6156):373-7. Epub 2013/10/19. doi: 10.1126/science.1241224. PubMed PMID: 24136970; PubMed Central PMCID: PMCPMC3880190.

26.Feng D, Liu T, Zhang X, Xiang T, Su W, Quan W, et al. Fingolimod improves diffuse brain injury by promoting AQP4 polarization and functional recovery of the glymphatic system. CNS Neurosci Ther. 2024;30(3):e14669. Epub 2024/03/09. doi: 10.1111/cns.14669. PubMed PMID: 38459666; PubMed Central PMCID: PMCPMC10924110.

27.Dong S, Zhao H, Nie M, Sha Z, Feng J, Liu M, et al. Cannabidiol Alleviates Neurological Deficits After Traumatic Brain Injury by Improving Intracranial Lymphatic Drainage. J Neurotrauma. 2024;41(15-16):e2009-e25. Epub 2024/03/30. doi: 10.1089/neu.2023.0539. PubMed PMID: 38553903.

28.Lv C, Han S, Sha Z, Liu M, Dong S, Zhang C, et al. Cerebral glucagon-like peptide-1 receptor activation alleviates traumatic brain injury by glymphatic system regulation in mice. CNS Neurosci Ther. 2023;29(12):3876-88. Epub 2023/06/24. doi: 10.1111/cns.14308. PubMed PMID: 37353947; PubMed Central PMCID: PMCPMC10651945.

29.Lu Q, Xiong J, Yuan Y, Ruan Z, Zhang Y, Chai B, et al. Minocycline improves the functional recovery after traumatic brain injury via inhibition of aquaporin-4. Int J Biol Sci. 2022;18(1):441-58. Epub 2022/01/04. doi: 10.7150/ijbs.64187. PubMed PMID: 34975343; PubMed Central PMCID: PMCPMC8692149.

30.Liu M, Huang J, Liu T, Yuan J, Lv C, Sha Z, et al. Exogenous interleukin 33 enhances the brain's lymphatic drainage and toxic protein clearance in acute traumatic brain injury mice. Acta Neuropathol Commun. 2023;11(1):61. Epub 2023/04/07. doi: 10.1186/s40478-023-01555-4. PubMed PMID: 37024941; PubMed Central PMCID: PMCPMC10080777.

31.Murdock MH, Yang CY, Sun N, Pao PC, Blanco-Duque C, Kahn MC, et al. Multisensory gamma stimulation promotes glymphatic clearance of amyloid. Nature. 2024;627(8002):149-56. Epub 2024/02/29. doi: 10.1038/s41586-024-07132-6. PubMed PMID: 38418876; PubMed Central PMCID: PMCPMC10917684 advisory board of Cognito Therapeutics. The other authors declare no competing interests.

32.Hussain R, Tithof J, Wang W, Cheetham-West A, Song W, Peng W, et al. Potentiating glymphatic drainage minimizes post-traumatic cerebral oedema. Nature. 2023;623(7989):992-1000. Epub 2023/11/16. doi: 10.1038/s41586-023-06737-7. PubMed PMID: 37968397; PubMed Central PMCID: PMCPMC11216305.

33.George KC, Kebejian L, Ruth LJ, Miller CW, Himelhoch S. Meta-analysis of the efficacy and safety of prazosin versus placebo for the treatment of nightmares and sleep disturbances in adults with posttraumatic stress disorder. J Trauma Dissociation. 2016;17(4):494-510. Epub 2016/02/03. doi: 10.1080/15299732.2016.1141150. PubMed PMID: 26835889.

34.Carney N, Totten AM, O'Reilly C, Ullman JS, Hawryluk GW, Bell MJ, et al. Guidelines for the Management of Severe Traumatic Brain Injury, Fourth Edition. Neurosurgery. 2017;80(1):6-15. Epub 2016/09/23. doi: 10.1227/NEU.0000000000001432. PubMed PMID: 27654000.

35.Pirker W, Katzenschlager R, Hallett M, Poewe W. Pharmacological Treatment of Tremor in Parkinson's Disease Revisited. J Parkinsons Dis. 2023;13(2):127-44. Epub 2023/02/28. doi: 10.3233/JPD-225060. PubMed PMID: 36847017; PubMed Central PMCID: PMCPMC10041452.

36.van der Heide A, Wessel M, Papadopetraki D, Geurts DEM, van Prooije TH, Gommans F, et al. Propranolol Reduces Parkinson's Tremor and Inhibits Tremor-Related Activity in the Motor Cortex: A Placebo-Controlled Crossover Trial. Ann Neurol. 2025;97(4):741-52. Epub 2024/12/21. doi: 10.1002/ana.27159. PubMed PMID: 39707791; PubMed Central PMCID: PMCPMC11889531.

37.Dominguez-Fernandez C, Kumar A, Kumar R, Darreh-Shori T. From heart to brain: cognitive potential of propranolol and diltiazem through cholinergic enhancement via butyrylcholinesterase inhibition. Front Pharmacol. 2025;16:1694610. Epub 2025/12/01. doi: 10.3389/fphar.2025.1694610. PubMed PMID: 41322300; PubMed Central PMCID: PMCPMC12657457.

38.Chai GS, Wu JJ, Gong J, Zhou JL, Jiang ZQ, Yi HY, et al. Activation of beta2-adrenergic Receptor Ameliorates Amyloid-beta-induced Mitophagy Defects and Tau Pathology in Mice. Neuroscience. 2022;505:34-50. Epub 2022/10/09. doi: 10.1016/j.neuroscience.2022.09.020. PubMed PMID: 36208707.

39.Sbardella D, Inzitari R, Iavarone F, Gioia M, Marini S, Sciandra F, et al. Enzymatic processing by MMP-2 and MMP-9 of wild-type and mutated mouse beta-dystroglycan. IUBMB life. 2012;64(12):988-94. doi: 10.1002/iub.1095. PubMed PMID: 23129308.

40.Waite A, Brown SC, Blake DJ. The dystrophin-glycoprotein complex in brain development and disease. Trends in neurosciences. 2012;35(8):487-96. doi: 10.1016/j.tins.2012.04.004. PubMed PMID: 22626542.

41.Mendelsohn AR, Larrick JW. Sleep facilitates clearance of metabolites from the brain: glymphatic function in aging and neurodegenerative diseases. Rejuvenation research. 2013;16(6):518-23. doi: 10.1089/rej.2013.1530. PubMed PMID: 24199995.
