# Supplementary material for: Propranolol improves cognitive function and glymphatic system recovery through STAT3/MMP2 pathway after TBI
Source: PLoS One. 2026 Aug 21;21(8):e0353852. doi: 10.1371/journal.pone.0353852 (PMC13496413; doi:10.1371/journal.pone.0353852)
Supplement: Supplementary file 3 [file pone.0353852.s004.docx]

Dear Reviewer:

Thank you for your valuable comments which are very helpful for revising and improving our paper. We have carefully studied your comments and made corresponding revision. Revised portion are marked in red in the submitted paper. The main corrections in the paper and the responds to the reviewer’s comments are as follows:

Reviewer’s comments:

Reviewer #2: This reviewers commends the authors for their extensive revision. The authors have significantly improved the scientific rigor of their study by adding the normal control group as suggested by the previous reviewer, and by expanding the discussion. The only part that requires additional minor revision is a thorough review of the manuscript by preferably a native English speaker to correct the numerous minor grammatical errors.

**Reply:** Thanks to you for your excellent comment. We tried our best to fix all the grammatical mistakes throughout the manuscript and fully complied with all the reviewer’s requirements.

Reviewer #3: First, the statistical comparisons are unclear. The manuscript should provide clear statistical comparisons between the sham vs. TBI groups and between the sham vs. propranolol-treated TBI groups where applicable. These comparisons are necessary to determine whether TBI significantly alters each measured outcome relative to baseline, and whether propranolol restores the outcome toward sham/control levels. In the figures, please clearly mention sham/control groups and as well as add statistical comparison with Sham/control groups
Second, Figures 2, 3, 4, and 6 do not include scale bars.
the study does not directly demonstrate that the STAT3/MMP2 pathway mediates propranolol’s effects. To support this mechanism, additional functional experiments would be needed. For example, the authors should test whether a STAT3 inhibitor mimics the effect of propranolol after TBI, whether MMP2 inhibition rescues AQP4 localization and glymphatic function, and whether MMP2 overexpression blocks or reduces the beneficial effects of propranolol.
Adding comparison with blocker will make it more strong.

**Reply:** Thanks to you for your excellent comment. First, all statistically significant comparisons were added to the Results and Figures. Second, scale bars were also added in Figures 2, 3, 4, and 6. Third, we introduced STAT3 and MMP2 inhibitors (S31-201 and SB-3CT) in vivo and vitro experiments. The results as:

**Result：**

**Propranolol improved cognitive function after TBI:**

Compared with untreated TBI rats, propranolol, S31-201 and SB-3CT all significantly relieved short- and long-term memory deficits in the novel object recognition and odor tests, though none of the three agents fully recovered memory function compared with the normal rats. In the Morris water maze test, propranolol, S31-201, and SB-3CT all alleviated the deficits in spatial learning and memory in TBI rats. However, none of these three agents fully restored spatial cognitive function compared with the normal rats. (Fig 1)

Figure1. A) The novel object recognition test revealed markedly improved short-term memory in the propranolol, S31-201 and SB-3CT treatment groups compared with the TBI group. Nevertheless, none of the three treatments fully rescued TBI-induced short-term memory deficits compared with normal rats. B) The odor test demonstrated obvious long-term memory improvements in the propranolol, S31-201 and SB-3CT treatment groups compared with the TBI group, whereas complete reversal of long-term memory impairment was not achieved relative to normal rats. C, D) In the Morris water maze test, TBI rats receiving propranolol, S31-201 or SB-3CT displayed significantly ameliorated spatial learning and memory capacities. Still, none of the interventions fully restored spatial cognitive function compared with normal controls. (*p<0.05, n=6 per group)

**Propranolol decreased p-tau/Aβ deposition and white matter (WM) damage:**

To explore the underlying mechanism by which propranolol improves cognitive function, we examined cerebral p-tau and Aβ deposition levels. The results demonstrated that propranolol, S31-201, and SB-3CT all totally reduced TBI-triggered p-tau and Aβ accumulation in the brain. Moreover, no statistically significant differences were detected among the three treatment groups and the normal group (Figure 2).

To further verify whether propranolol, S31-201, and SB-3CT could rescue TBI-induced white matter (WM) integrity damage, brain myelin density was quantified. As illustrated in Figure 2, myelin density was markedly restored in rats treated with propranolol, S31-201, or SB-3CT relative to untreated TBI rats. Nevertheless, none of the three interventions fully restored myelin density to the level observed in normal rats.

Figure2. (A–F) Propranolol, S31-201, and SB-3CT all totally alleviated TBI-induced cerebral p-tau accumulation. No statistically significant differences were observed among the three treatment groups and the normal control group. G–L) Similarly, propranolol, S31-201, and SB-3CT totally lowered TBI-triggered brain Aβ accumulation, with no significant differences detected among the treatment groups and normal controls. (M–R) Myelin density was significantly recovered in drug-treated rats compared with untreated TBI rats. However, none of the three interventions restored myelin density completely to the level of normal rats. (*p<0.05, n=6/group,10×40 objective lens).

**Propranolol recovered** **glymphatic dysfunction in TBI rats:**

Since propranolol was found to reduce cerebral p-tau and Aβ deposition following TBI, we further examined glymphatic system function. The results revealed that propranolol, S31-201, and SB-3CT totally rescued TBI-induced delay in metabolic waste clearance. No significant differences were detected among the three treatment groups and the normal group. (Fig3)

Figure3. (A-F) Propranolol, S31-201 and SB-3CT totally rescued TBI-induced delay in metabolic waste clearance. No significant differences were detected among the three treatment groups and the normal group. (*p<0.05, n=5/group)

**Propranolol restored AQP4 expression after TBI:**

To elucidate the mechanism by which propranolol ameliorates TBI-induced impairment of glymphatic function, we quantified perivascular AQP4 expression and astrocyte levels in the brain. The results demonstrated that propranolol, S31-201, and SB-3CT totally restored perivascular and astrocytic AQP4 expression relative to untreated TBI rats. No statistically significant differences were identified among the three treatment groups and the normal group. (Fig 4)

Figure4. (A–C, G–I, M–O, S–U, Y–a, e) Propranolol, S31-201, and SB-3CT totally restored perivascular AQP4 (green) expression compared with untreated TBI rats. No statistically significant differences were detected among the three treatment groups and the normal group. Blood vessels were labeled with vWF (von Willebrand factor, red), and cell nuclei were counterstained with DAPI. (D–F, J–L, P–R, V–X, b–d, f) Propranolol, S31-201, and SB-3CT totally restored astrocytic AQP4 (green) expression compared with untreated TBI rats. No statistically significant differences were detected among the three treatment groups and the normal group. Astrocytes were labeled with glial fibrillary acidic protein (GFAP, red), and cell nuclei were counterstained with DAPI. (*p<0.05, n=6/group, 10×40 objective lens)

**Propranolol decreased p-STAT3/MMP2 expression after TBI:**

To further explore the mechanism of propranolol-induced improvement of cognitive function and glymphatic system recovery after TBI, Western blot was used to test the expression of phosphorylated STAT3 (p-STAT3) and MMP2. The results showed that the levels of p-STAT3 and MMP2 were significantly downregulated in the propranolol-treated TBI rats, but propranolol could not completely reverse the increased levels of p-STAT3 and MMP2 after TBI compared with normal rats. (Fig5)

**Propranolol reversed the noradrenaline-mediated increased expression of p-STAT3/MMP2** **in astrocytes:**

To further clarify the mechanism of propranolol-induced reduction in p-STAT3/MMP2 expression in TBI rats, we treated astrocytes with noradrenaline and propranolol. Western blot data showed that the levels of p-STAT3 and MMP2 were upregulated after astrocytes were incubated with noradrenaline. Subsequent addition of propranolol reversed the noradrenaline-induced elevation in p-STAT3 and MMP2 expression; however, propranolol failed to fully restore p-STAT3 and MMP2 levels to those of the control group. No significant difference was observed between the propranolol-only group and the control group. (Fig5)

Figure5. A, B) MMP2 protein level was markedly decreased in propranolol-treated rats compared with TBI model rats. Nevertheless, propranolol could not fully restore MMP2 upregulation caused by TBI to the level of normal rats. GAPDH was applied as the loading reference. A, C) Western blot quantification demonstrated that p-STAT3 expression was significantly lower in the propranolol intervention group than in the TBI group, whereas complete recovery of TBI-induced abnormal p-STAT3 expression was not achieved by propranolol treatment relative to normal rats. Total STAT3 was set as the loading control. D, E) Incubation with noradrenaline led to significant upregulation of MMP2 in astrocytes. Additional propranolol treatment rescued the noradrenaline-mediated elevation in MMP2 expression, though full normalization to the control group level was not attained. No statistical difference was detected between the single propranolol group and the control group. GAPDH served as the loading control. D, F) Noradrenaline stimulation significantly increased p-STAT3 levels in astrocytes, and this upregulation was partially counteracted by propranolol supplementation. However, p-STAT3 expression could not be fully restored to the baseline level of the control group. No statistical difference was observed between the single propranolol group and the control group. Total STAT3 was used as the loading control. (*p < 0.05)

**Propranolol antagonized the noradrenaline-mediated decline expression of AQP4 of astrocytes:**

To further elucidate how propranolol elevates AQP4 expression following TBI, astrocytes were subjected to treatments with noradrenaline alone or combined with propranolol, S31-201 or SB-3CT. Immunohistochemical staining was utilized to quantify AQP4 expression in astrocytes. Noradrenaline treatment markedly downregulated AQP4 levels in astrocytes, whereas co-administration of propranolol, S31-201 or SB-3CT fully rescued this noradrenaline-triggered AQP4 downregulation. (Fig6)

Figure6 D–F, S) AQP4 (red) expression was significantly decreased in astrocytes following noradrenaline treatment relative to the control group. G–I, S) Treatment with propranolol fully restored the noradrenaline-evoked downregulation of AQP4 (red) in astrocytes. J–L, S) No statistically significant difference in AQP4 (red) expression was detected between the propranolol single-treatment group and the control group. M–O, S) S31-201 fully rescued the noradrenaline-induced reduction in astrocytic AQP4 (red) expression. P–R, S) SB-3CT also completely reversed the noradrenaline-triggered decline in AQP4 (red) expression in astrocytes. Nuclei were counterstained with DAPI. *p<0.05, 10×20 objective lens, 10 of fields and 6 of cells per field were counted).

Reviewer #4: 1. The authors have comprehensively addressed the three major issues raised in the previous round. They have added a normal healthy control group to differentiate TBI pathology from drug effects, provided precision and stereotaxic instrumentation details for the glymphatic system detection method, and extensively expanded their discussion to integrate broader clinical frameworks, limitations, and relevant neurological disease models.

2. By incorporating a healthy normal rat control group alongside the TBI and TBI + Propranolol groups, the in vivo experimental structure is now complete and rigorous. The added findings confirm that while propranolol significantly improves spatial learning, memory, and white matter integrity, it does not completely reverse all TBI deficits compared to healthy controls, which demonstrates a realistic and data-supported conclusion. The matching in vitro model using astrocytes treated with norepinephrine further confirms the mechanistic role of the STAT3/MMP2 pathway.

3. The statistical evaluation is robust. An investigator blinded to the experimental groupings conducted the quantification. Multiple brain slices (five slides per sample, eight fields per slide) were digitized uniformly via an objective MCID image analysis system using density thresholds to eliminate bias. Student’s t-test was appropriately utilized to evaluate variations between distinct groups, and data are standardly presented as mean ± SE.

4.The authors state that they have added the missing statistical baselines and control configurations directly into the main text and figures (e.g., Figure 1 revisions) to ensure compliance with PLOS data sharing policies. (Note: Please do a quick final check of the full manuscript PDF to confirm no separate raw data repository was omitted).

5. The revision reads clearly and follows standard scientific English formatting. The expanded text and author replies are organized logically. A minor typographical error exists on Page 2 / Page 6 where the section thickness is written as "80 mm thick sections"—this should be corrected to micrometers (µm) as seen elsewhere in the histology protocol.

**Reply:** Thanks to you for your good comment. The "80 mm thick sections" was corrected to micrometers (µm).
