# Supplementary material for: Propranolol improves cognitive function and glymphatic system recovery through STAT3/MMP2 pathway after TBI
Source: PLoS One. 2026 Aug 21;21(8):e0353852. doi: 10.1371/journal.pone.0353852 (PMC13496413; doi:10.1371/journal.pone.0353852)
Supplement: Peer review history

# Peer review: Decision Letter 0

**DOI:** 10.1371/journal.pone.0353852.r001

**Authors:** Firas Kobeissy

9 Oct 2025

Dear Dr. yu,

Thank you for submitting your manuscript to PLOS ONE. After careful consideration, we feel that it has merit but does not fully meet PLOS ONE’s publication criteria as it currently stands. Therefore, we invite you to submit a revised version of the manuscript that addresses the points raised during the review process.

Please submit your revised manuscript by Nov 23 2025 11:59PM. If you will need more time than this to complete your revisions, please reply to this message or contact the journal office at plosone@plos.org. When you're ready to submit your revision, log on to https://www.editorialmanager.com/pone/ and select the 'Submissions Needing Revision' folder to locate your manuscript file.

- A rebuttal letter that responds to each point raised by the academic editor and reviewer(s). You should upload this letter as a separate file labeled 'Response to Reviewers'.
- A marked-up copy of your manuscript that highlights changes made to the original version. You should upload this as a separate file labeled 'Revised Manuscript with Track Changes'.
- An unmarked version of your revised paper without tracked changes. You should upload this as a separate file labeled 'Manuscript'.

If you would like to make changes to your financial disclosure, please include your updated statement in your cover letter. Guidelines for resubmitting your figure files are available below the reviewer comments at the end of this letter.

If applicable, we recommend that you deposit your laboratory protocols in protocols.io to enhance the reproducibility of your results. Protocols.io assigns your protocol its own identifier (DOI) so that it can be cited independently in the future. For instructions see: https://journals.plos.org/plosone/s/submission-guidelines#loc-laboratory-protocols. Additionally, PLOS ONE offers an option for publishing peer-reviewed Lab Protocol articles, which describe protocols hosted on protocols.io. Read more information on sharing protocols at https://plos.org/protocols?utm_medium=editorial-email&utm_source=authorletters&utm_campaign=protocols.

We look forward to receiving your revised manuscript.

Kind regards,

Firas H Kobeissy, PhD

Academic Editor

PLOS ONE

Journal Requirements:

When submitting your revision, we need you to address these additional requirements.

1. Please ensure that your manuscript meets PLOS ONE's style requirements, including those for file naming. The PLOS ONE style templates can be found at

https://journals.plos.org/plosone/s/file?id=wjVg/PLOSOne_formatting_sample_main_body.pdf and https://journals.plos.org/plosone/s/file?id=ba62/PLOSOne_formatting_sample_title_authors_affiliations.pdf

2. To comply with PLOS ONE submissions requirements, in your Methods section, please provide additional information regarding the experiments involving animals and ensure you have included details on (1) methods of sacrifice, (2) methods of anesthesia and/or analgesia, and (3) efforts to alleviate suffering.

3. Thank you for stating in your Funding Statement:

[The work is supported by the National Natural Science Foundation of China (grant Nos. 81801217, 81801215)].

Please provide an amended statement that declares *all* the funding or sources of support (whether external or internal to your organization) received during this study, as detailed online in our guide for authors at http://journals.plos.org/plosone/s/submit-now.  Please also include the statement “There was no additional external funding received for this study.” in your updated Funding Statement.

Please include your amended Funding Statement within your cover letter. We will change the online submission form on your behalf.

4. Thank you for stating the following in your manuscript:

[The work is supported by the National Natural Science Foundation of China (grant Nos. 81801217, 81801215)]

We note that you have provided funding information that is currently declared in your Funding Statement. However, funding information should not appear in the Acknowledgments section or other areas of your manuscript. We will only publish funding information present in the Funding Statement section of the online submission form.

Please remove any funding-related text from the manuscript and let us know how you would like to update your Funding Statement. Currently, your Funding Statement reads as follows:

[The work is supported by the National Natural Science Foundation of China (grant Nos. 81801217, 81801215)]

Please include your amended statements within your cover letter; we will change the online submission form on your behalf.

5. We note that the grant information you provided in the ‘Funding Information’ and ‘Financial Disclosure’ sections do not match.

When you resubmit, please ensure that you provide the correct grant numbers for the awards you received for your study in the ‘Funding Information’ section.

6. We note that your Data Availability Statement is currently as follows: [All relevant data are within the manuscript and its Supporting Information files.]

Please confirm at this time whether or not your submission contains all raw data required to replicate the results of your study. Authors must share the “minimal data set” for their submission. PLOS defines the minimal data set to consist of the data required to replicate all study findings reported in the article, as well as related metadata and methods (https://journals.plos.org/plosone/s/data-availability#loc-minimal-data-set-definition).

For example, authors should submit the following data:

- The values behind the means, standard deviations and other measures reported;

- The values used to build graphs;

- The points extracted from images for analysis.

Authors do not need to submit their entire data set if only a portion of the data was used in the reported study.

If your submission does not contain these data, please either upload them as Supporting Information files or deposit them to a stable, public repository and provide us with the relevant URLs, DOIs, or accession numbers. For a list of recommended repositories, please see https://journals.plos.org/plosone/s/recommended-repositories.

If there are ethical or legal restrictions on sharing a de-identified data set, please explain them in detail (e.g., data contain potentially sensitive information, data are owned by a third-party organization, etc.) and who has imposed them (e.g., an ethics committee). Please also provide contact information for a data access committee, ethics committee, or other institutional body to which data requests may be sent. If data are owned by a third party, please indicate how others may request data access.

7. PLOS ONE now requires that authors provide the original uncropped and unadjusted images underlying all blot or gel results reported in a submission’s figures or Supporting Information files. This policy and the journal’s other requirements for blot/gel reporting and figure preparation are described in detail at https://journals.plos.org/plosone/s/figures#loc-blot-and-gel-reporting-requirements and https://journals.plos.org/plosone/s/figures#loc-preparing-figures-from-image-files. When you submit your revised manuscript, please ensure that your figures adhere fully to these guidelines and provide the original underlying images for all blot or gel data reported in your submission. See the following link for instructions on providing the original image data: https://journals.plos.org/plosone/s/figures#loc-original-images-for-blots-and-gels.

In your cover letter, please note whether your blot/gel image data are in Supporting Information or posted at a public data repository, provide the repository URL if relevant, and provide specific details as to which raw blot/gel images, if any, are not available. Email us at plosone@plos.org if you have any questions.

8. Please ensure that you include a title page within your main document. You should list all authors and all affiliations as per our author instructions and clearly indicate the corresponding author.

9. Please include your full ethics statement in the ‘Methods’ section of your manuscript file. In your statement, please include the full name of the IRB or ethics committee who approved or waived your study, as well as whether or not you obtained informed written or verbal consent. If consent was waived for your study, please include this information in your statement as well.

If the reviewer comments include a recommendation to cite specific previously published works, please review and evaluate these publications to determine whether they are relevant and should be cited. There is no requirement to cite these works unless the editor has indicated otherwise.

[Note: HTML markup is below. Please do not edit.]

Reviewers' comments:

Reviewer's Responses to Questions

**Comments to the Author**

1. Is the manuscript technically sound, and do the data support the conclusions?

Reviewer #1: Yes

**********

2. Has the statistical analysis been performed appropriately and rigorously?

Reviewer #1: Yes

**********

3. Have the authors made all data underlying the findings in their manuscript fully available?

The PLOS Data policy

Reviewer #1: Yes

**********

4. Is the manuscript presented in an intelligible fashion and written in standard English?

Reviewer #1: Yes

**********

Reviewer #1: This manuscript innovatively proposes a hypothesis that propranolol may regulate the function of the lymphoid system through the STAT3/MMP2 pathway to improve cognition, and this hypothesis is verified through in vitro and in vivo experiments. The research design is logically coherent, and the technical route is clear. It provides a new mechanism explanation and potential therapeutic direction for the repair of cognitive function after TBI, and has certain scientific value and clinical reference significance. However, the research still has areas that can be improved in terms of experimental design details, mechanism depth, and result interpretation. Further supplementation and optimization are required.

1. Animal Experiment Design

Group Completeness: The study only sets up the TBI group and the TBI + propranolol group, lacking a normal control group (healthy rats that did not undergo TBI surgery). The absence of the normal control group makes it impossible to clearly determine the effect of propranolol on the cognitive function, lymphoid system, and related protein expression of normal rats, and also makes it difficult to completely distinguish the pathological damage of TBI itself from the specific effect of propranolol intervention. It is recommended to supplement the normal control group to more comprehensively evaluate the effect of propranolol.

2. Detection Method Details

Lymphoid System Function Detection: The method of injecting 70kD dextran into the cerebellopontine cistern and detecting fluorescence density is used to evaluate the lymphoid system function. However, the accuracy control measures for the injection site (such as whether it is precisely located by a stereotactic instrument and whether there is a record of the injection position after the injection for anatomical verification) are not explained. Deviation in the injection site may lead to uneven distribution of dextran and affect the reliability of the quantitative results of fluorescence density. It is recommended to supplement the experimental details of precise control of the injection site.

3. Discussion and Conclusion

The discussion only compares the differences between propranolol and other β receptor blockers (such as lipophilicity), but does not fully combine the recent research progress on the regulation of the lymphoid system after TBI (such as the effects of other drugs and physical intervention methods on the lymphoid system), nor discusses the similarities and differences between the results of this study and other studies on the role of propranolol in neurological diseases (such as the research conclusions of propranolol in Alzheimer's disease and Parkinson's disease). It is recommended to expand the discussion scope, place this study in a broader research context, and highlight the innovation and limitations of the study.,This manuscript innovatively proposes a hypothesis that propranolol may regulate the function of the lymphoid system through the STAT3/MMP2 pathway to improve cognition, and this hypothesis is verified through in vitro and in vivo experiments. The research design is logically coherent, and the technical route is clear. It provides a new mechanism explanation and potential therapeutic direction for the repair of cognitive function after TBI, and has certain scientific value and clinical reference significance. However, the research still has areas that can be improved in terms of experimental design details, mechanism depth, and result interpretation. Further supplementation and optimization are required.

1. Animal Experiment Design

Group Completeness: The study only sets up the TBI group and the TBI + propranolol group, lacking a normal control group (healthy rats that did not undergo TBI surgery). The absence of the normal control group makes it impossible to clearly determine the effect of propranolol on the cognitive function, lymphoid system, and related protein expression of normal rats, and also makes it difficult to completely distinguish the pathological damage of TBI itself from the specific effect of propranolol intervention. It is recommended to supplement the normal control group to more comprehensively evaluate the effect of propranolol.

2. Detection Method Details

Lymphoid System Function Detection: The method of injecting 70kD dextran into the cerebellopontine cistern and detecting fluorescence density is used to evaluate the lymphoid system function. However, the accuracy control measures for the injection site (such as whether it is precisely located by a stereotactic instrument and whether there is a record of the injection position after the injection for anatomical verification) are not explained. Deviation in the injection site may lead to uneven distribution of dextran and affect the reliability of the quantitative results of fluorescence density. It is recommended to supplement the experimental details of precise control of the injection site.

3. Discussion and Conclusion

The discussion only compares the differences between propranolol and other β receptor blockers (such as lipophilicity), but does not fully combine the recent research progress on the regulation of the lymphoid system after TBI (such as the effects of other drugs and physical intervention methods on the lymphoid system), nor discusses the similarities and differences between the results of this study and other studies on the role of propranolol in neurological diseases (such as the research conclusions of propranolol in Alzheimer's disease and Parkinson's disease). It is recommended to expand the discussion scope, place this study in a broader research context, and highlight the innovation and limitations of the study.

**********

what does this mean?). If published, this will include your full peer review and any attached files.

If you choose “no”, your identity will remain anonymous but your review may still be made public.

**Do you want your identity to be public for this peer review?**  For information about this choice, including consent withdrawal, please see our Privacy Policy

Reviewer #1: No

**********

[NOTE: If reviewer comments were submitted as an attachment file, they will be attached to this email and accessible via the submission site. Please log into your account, locate the manuscript record, and check for the action link "View Attachments". If this link does not appear, there are no attachment files.]

While revising your submission, please upload your figure files to the Preflight Analysis and Conversion Engine (PACE) digital diagnostic tool, https://pacev2.apexcovantage.com/. PACE helps ensure that figures meet PLOS requirements. To use PACE, you must first register as a user. Registration is free. Then, login and navigate to the UPLOAD tab, where you will find detailed instructions on how to use the tool. If you encounter any issues or have any questions when using PACE, please email PLOS at figures@plos.org. Please note that Supporting Information files do not need this step.

# Author comment: Author response to Decision Letter 1

**DOI:** 10.1371/journal.pone.0353852.r002

20 Jan 2026

Dear Reviewer:

Thank you for your valuable comments which are very helpful for revising and improving our paper. We have carefully studied your comments and made corresponding revision. Revised portion are marked in red in the submitted paper. The main corrections in the paper and the responds to the reviewer’s comments are as follows:

Reviewer’s comments:

1. Animal Experiment Design

Group Completeness: The study only sets up the TBI group and the TBI + propranolol group, lacking a normal control group (healthy rats that did not undergo TBI surgery). The absence of the normal control group makes it impossible to clearly determine the effect of propranolol on the cognitive function, lymphoid system, and related protein expression of normal rats, and also makes it difficult to completely distinguish the pathological damage of TBI itself from the specific effect of propranolol intervention. It is recommended to supplement the normal control group to more comprehensively evaluate the effect of propranolol.

Reply: Thanks to you for your excellent comment. We added normal control group (healthy rats that did not undergo TBI surgery) in our study. It turned out that even though propranolol could improve cognitive function after TBI, propranolol could not completely reverse the short-term memory, long-term memory and spatial learning deficits after TBI as compared to normal rats. On the other hand, propranolol could totally reverse TBI-induced p-tau/Aβ deposition. Myelin densities were significantly recovered in the brains of propranolol-treated rats, but propranolol could not completely reverse the myelin densities deficits after TBI as compared to normal rats. Also, propranolol could completely reverse the glymphatic dysfunction after TBI. Moreover, propranolol totally recovered the AQP4 accumulation around astrocyte and vessels after TBI as compared to normal group. The results showed that the level of p-STAT3/MMP2 were significantly downregulated in the propranolol-treated rats, but propranolol could not completely reverse the increased level of p-STAT3/MMP2 after TBI as compared to normal rats. In vitro, it turned out that propranolol reversed the noradrenaline-mediated increased expression of p-STAT3/MMP2 in astrocytes, but propranolol could not totally restore the noradrenaline-mediated expression of p-STAT3/MMP2 as compared to control group. We found that noradrenaline could decrease the lever of AQP4 expression of astrocytes. On the other hand, it showed that propranolol totally reversed the noradrenaline-mediated decline expression of AQP4 of astrocytes.

2. Detection Method Details

Lymphoid System Function Detection: The method of injecting 70kD dextran into the cerebellopontine cistern and detecting fluorescence density is used to evaluate the lymphoid system function. However, the accuracy control measures for the injection site (such as whether it is precisely located by a stereotactic instrument and whether there is a record of the injection position after the injection for anatomical verification) are not explained. Deviation in the injection site may lead to uneven distribution of dextran and affect the reliability of the quantitative results of fluorescence density. It is recommended to supplement the experimental details of precise control of the injection site.

Reply: Thanks to you for your excellent comment. The detail of Lymphoid System Function Detection was added as follow: Anesthetized rats were fixed in a stereotaxic frame and a 30GA needle was inserted into the central cisterna magna to avoid uneven distribution of dextran. 5µl of Dextran (Sigma, MW: 70 kD, USA) was injected into the cisterna magna of TBI rats over 5 minutes using a syringe pump at a flow rate of 2μl/min. Rats were sacrificed at 30 minutes from the start infusion. The rats were transcardially perfused with 0.9% saline followed by 4% paraformaldehyde. 80 mm thick sections were cut and imaged using a 10x objective of a laser-scanning confocal microscope (Zeiss LSM 510 NLO, Carl Zeiss, Germany). MCID image analysis system (Imaging Research, St. Catharines, Ontario, Canada) was used to quantify the fluorescence density. The data were presented as percentage of positive fluorescence area in the brain cross section.

3. Discussion and Conclusion

The discussion only compares the differences between propranolol and other β receptor blockers (such as lipophilicity), but does not fully combine the recent research progress on the regulation of the lymphoid system after TBI (such as the effects of other drugs and physical intervention methods on the lymphoid system), nor discusses the similarities and differences between the results of this study and other studies on the role of propranolol in neurological diseases (such as the research conclusions of propranolol in Alzheimer's disease and Parkinson's disease). It is recommended to expand the discussion scope, place this study in a broader research context, and highlight the innovation and limitations of the study.,This manuscript innovatively proposes a hypothesis that propranolol may regulate the function of the lymphoid system through the STAT3/MMP2 pathway to improve cognition, and this hypothesis is verified through in vitro and in vivo experiments. The research design is logically coherent, and the technical route is clear. It provides a new mechanism explanation and potential therapeutic direction for the repair of cognitive function after TBI, and has certain scientific value and clinical reference significance. However, the research still has areas that can be improved in terms of experimental design details, mechanism depth, and result interpretation. Further supplementation and optimization are required.

Reply: Thanks to you for your good comment. As reviewer suggested, we expanded the discussion scope. The changed discussion section was labeled with red in the main manuscript.

Discussion:

TBI could cause a series of symptoms, such as declined cognitive function, headaches, depression, fatigue, irritability, and anxiety, which influences living quality of TBI patients [1]. Multiple TBI patients benefit from administration of β-blockers (BBs). As one of BBs, propranolol may have more benefits than other BBs because of the lipophilic properties, increasing cerebral perfusion and decreasing catabolism and oxygen consumption of brain [20]. In this research, we conformed that propranolol could enhance defective cognitive function after TBI. But, the mechanism of propranolol-induced cognitive function recovery is still unknown.

These are numbers of proteinaceous waste products (for example: p-tau and Aβ etc.) deposition in chronic traumatic encephalopathy (CTE) and sleep disruption patients after TBI. The waste products are thought to contribute to neurodegeneration processes as Alzheimer’s disease (AD) and TBI [21]. The glymphatic system which helps to clear the waste products in brain is a promising treatment target after TBI. There are lots of factors that could influence the function of glymphatic system, like astrocyte dysfunction and perivascular spaces (PVS) disruption after TBI [22]. AQP4 is wildly distributed in capillary endothelial cells, astrocytes and ependymocytes in the brain. The level of AQP4 is significantly downregulated at 6h after TBI. The cerebral contusion is usually accompanied with cerebral edema, which takes responsible for a variety of syndromes after TBI. It has showed that the TBI-induced edema is deteriorated in the AQP4 knock-out rat which suggested that AQP4 plays an essential role in edema after TBI [23]. Meanwhile, the expression of AQP4 of vessels and astrocytes play an essential role in glymphatic system [8]. Thus, due to the ubiquitous heterogeneity of individuals, conditions, and pathophysiological mechanisms after TBI [24], treating glymphatic system dysfunction maybe a common pathway underlying these variations. The potential TBI treatment targeted at glymphatic system has made big strides these years. Sleep recovery may relieve TBI-induced cognitive impairment. Because, natural sleep or anesthesia could increase convective exchange of cerebrospinal fluid with interstitial fluid which may help to increase the rate of β-amyloid clearance[25]. Administering fingolimod could treat TBI effectively by promoting AQP4 repolarization and improving glymphatic system function [26]. Similar to fingolimod, cannabidiol, exendin-4, minocycline and interleukin-33 could all recover the function of TBI through regulating AQP4 polarization or increasing the AQP4 expression [27-30]. Multisensory gamma stimulation (40 Hz stimulation) can restore glymphatic system to clear amyloid by enhancing the polarization of AQP4, increasing the volume of meningeal lymphatic vessels (mLVs) and synchronizing multiple cells in glymphatic flow [31]. Previous studies suggest that adrenergic inhibitors promote fluid drainage from brain tissue, alleviate sleep disturbance, and decrease brain edema [32, 33]. Thus, it seems that adrenergic inhibitors, like propranolol, have a strong relationship with glymphatic system. In this study, we unexpectedly found that propranolol decreased p-tau and Aβ deposition in brain by glymphatic system recovery after TBI. The results also revealed the facts that propranolol could restore the expression of AQP4 on cerebral blood vessels and astrocytes, which may partly explain the mechanism of propranolol-induced glymphatic system recovery. To sum up, Our and previous studies suggest that AQP4 is a common regulated target that influencing the glymphatic system for the most of treating TBI drugs. Moreover, Other clinical studies have found other considerable beneficial effects of adrenergic inhibition, such as alleviating sleep disturbance [33], improving psychiatric disorders [32]. Thus, adrenergic inhibition, like propranolol, may reveal more diversified beneficial effects than other treating TBI drugs that targeted glymphatic system only. But, the clinical translation of adrenergic inhibition has been challenging, because maintaining cerebral blood perfusion is critical for the TBI patients according to existing guidelines [34]. Thus, this conflict requires further basic and clinical research to resolve.

Propranolol also has significant relationship with other neurological diseases. These are studies show that propranolol could reduce Parkinson’s disease (PD) tremor effectively [35, 36]. The effect of propranolol is may influenced by beta-1 adrenergic receptor in the motor cortex.[36] Also, propranolol represents promising therapeutic effects in Alzheimer's disease (AD) and other cognitive disorders through cholinergic enhancement via butyrylcholinesterase inhibition.[37] In the contrary, there is study shown that the activation of beta-2 adrenergic receptor ameliorated amyloid-β-induced memory deficits. Inhibition of beta-2 adrenergic receptor by propranolol blocks the activation of beta-2 adrenergic receptor-induced improvements in pathological changes [38]. Thus, what role of propranolol is played in the neurological diseases is still controversial. More basic and clinical researches are also needed to explore the mechanism of propranolol for the future.

Hence, we further explored what mechanism induced propranolol-mediated AQP4 recovery of TBI. Dystroglycan (DG) widely expresses at the interface between the basement membrane and the cell membrane in various different kinds of tissues. DG plays an essential role in for maintaining the polarization of AQP4 in astrocytes. There are evidences indicate that nonspecific matrix metalloproteinase (MMP) inhibitors reverse the level of DG in astrocyte under oxygen–glucose deprivation condition, which implies that MMP takes responsible for the astrocyte-induced DG reduction. There are evidences show that MMP-2 is involved in cleavage of DG in cerebral ischemia, but the mechanism remains unclear [10, 39, 40]. Norepinephrine (NE), as a neurotransmitter and neuromodulator, plays a major role in multiple activities of neuronal or non-neuronal cells. The evidences show that NE promotes the expression of phosphorylated signal transducer and activator of transcription 3 (p-STAT3) dependent on a concentration-dependent manner in pancreatic cancer cells. NE-induced phosphorylation of STAT3 is inhibited by propranolol (aβ-adrenergic receptor inhibitor, β-ARI). Also, the level of MMP2 is downregulated through blocking STAT3 [11]. Interestingly, it is reported that clearance rate of glymphatic system is faster during sleep than during waking hours [41]. In the brain, the NE-the locus coeruleus (LC) system is shut down during sleep which may help to potentate more rapid clearance of waste products [21]. Thus, we speculated that NE/β-ARI/STAT3 signaling pathway may take responsible for deficiency of AQP4 after TBI. In the present study, we found that propranolol decreased p-STAT3/MMP2 expression after TBI in vivo which may partly explain the mechanism of propranolol-mediated AQP4 recovery of TBI. The vitro research showed that propranolol reversed the noradrenaline-mediated increased expression of p-STAT3/MMP2 and decline expression of AQP4 of astrocytes, which further confirmed our hypothesis.

Our study provides a new mechanism explanation and potential therapeutic direction of propranolol for the repair of cognitive function after TBI. But there are still lots of limitations in our study. Even though, we confirmed that propranolol improved cognitive function after TBI. But we could not explain why propranolol could not completely reverse the memory deficits, myelin densities deficits and expression of p-STAT3/MMP2 after TBI as compared to normal rats. Moreover, in our study (data not seen), high dose of propranolol (10mg/kg) may harm the cognitive function after TBI. Thus, we hypothesize if there are other mechanisms of propranolol-mediated benefits or disadvantages. As shown in other study, propranolol played an important role in regulating inflammation which was not seen in our study. There are different adrenergic receptors (α and β) in multiple types of cells in the brain. What roles of differential adrenergic receptors are played in TBI is still need further explored. Whether propranolol could influence AQP4 polarization directly still need to be further explored [7].

Thank you for your constructive comments. We tried our best to improve the manuscript and made some changes in the manuscript. These changes will not influence the content and framework of the paper. We appreciate for Reviewer’ warm work earnestly, and hope that the correction will meet with approval.

Once again, thank you very much for your comments and suggestions.

References�

1.McInnes K, Friesen CL, MacKenzie DE, Westwood DA, Boe SG. Correction: Mild Traumatic Brain Injury (mTBI) and chronic cognitive impairment: A scoping review. PloS one. 2019;14(6):e0218423. doi: 10.1371/journal.pone.0218423. PubMed PMID: 31185044; PubMed Central PMCID: PMC6559670.

2.Ramos-Cejudo J, Wisniewski T, Marmar C, Zetterberg H, Blennow K, de Leon MJ, et al. Traumatic Brain Injury and Alzheimer's Disease: The Cerebrovascular Link. EBioMedicine. 2018;28:21-30. doi: 10.1016/j.ebiom.2018.01.021. PubMed PMID: 29396300; PubMed Central PMCID: PMC5835563.

3.Iliff JJ, Chen MJ, Plog BA, Zeppenfeld DM, Soltero M, Yang L, et al. Impairment of glymphatic pathway function promotes tau pathology after traumatic brain injury. The Journal of neuroscience : the official journal of the Society for Neuroscience. 2014;34(49):16180-93. doi: 10.1523/JNEUROSCI.3020-14.2014. PubMed PMID: 25471560; PubMed Central PMCID: PMC4252540.

4.Khalili H, Ahl R, Paydar S, Sjolin G, Cao Y, Abdolrahimzadeh Fard H, et al. Beta-Blocker Therapy in Severe Traumatic Brain Injury: A Prospective Randomized Controlled Trial. World journal of surgery. 2020;44(6):1844-53. doi: 10.1007/s00268-020-05391-8. PubMed PMID: 32002583.

5.Solito E, Sastre M. Microglia function in Alzheimer's disease. Frontiers in pharmacology. 2012;3:14. doi: 10.3389/fphar.2012.00014. PubMed PMID: 22363284; PubMed Central PMCID: PMC3277080.

6.Acosta C, Anderson HD, Anderson CM. Astrocyte dysfunction in Alzheimer disease. Journal of neuroscience research. 2017;95(12):2430-47. doi: 10.1002/jnr.24075. PubMed PMID: 28467650.

7.O'Donnell J, Zepp

Attachment Submitted filename: Responses to reviewer.docx

# Peer review: Decision Letter 1

**DOI:** 10.1371/journal.pone.0353852.r003

**Authors:** Firas Kobeissy

21 May 2026

Dear Dr. yu,

Thank you for submitting your manuscript to PLOS ONE. After careful consideration, we feel that it has merit but does not fully meet PLOS ONE’s publication criteria as it currently stands. Therefore, we invite you to submit a revised version of the manuscript that addresses the points raised during the review process.

Please submit your revised manuscript by Jul 05 2026 11:59PM. If you will need more time than this to complete your revisions, please reply to this message or contact the journal office at plosone@plos.org. When you're ready to submit your revision, log on to https://www.editorialmanager.com/pone/ and select the 'Submissions Needing Revision' folder to locate your manuscript file.

- A letter that responds to each point raised by the academic editor and reviewer(s). You should upload this letter as a separate file labeled 'Response to Reviewers'.
- A marked-up copy of your manuscript that highlights changes made to the original version. You should upload this as a separate file labeled 'Revised Manuscript with Track Changes'.
- An unmarked version of your revised paper without tracked changes. You should upload this as a separate file labeled 'Manuscript'.

If you would like to make changes to your financial disclosure, please include your updated statement in your cover letter. Guidelines for resubmitting your figure files are available below the reviewer comments at the end of this letter.

If applicable, we recommend that you deposit your laboratory protocols in protocols.io to enhance the reproducibility of your results. Protocols.io assigns your protocol its own identifier (DOI) so that it can be cited independently in the future. For instructions see: https://journals.plos.org/plosone/s/submission-guidelines#loc-laboratory-protocols. Additionally, PLOS ONE offers an option for publishing peer-reviewed Lab Protocol articles, which describe protocols hosted on protocols.io. Read more information on sharing protocols at https://plos.org/protocols?utm_medium=editorial-email&utm_source=authorletters&utm_campaign=protocols.

As the corresponding author, your ORCID iD is verified in the submission system and will appear in the published article. PLOS supports the use of ORCID, and we encourage all coauthors to register for an ORCID iD and use it as well. Please encourage your coauthors to verify their ORCID iD within the submission system before final acceptance, as unverified ORCID iDs will not appear in the published article. *Only*  the individual author can complete the verification step; PLOS staff *cannot*  verify ORCID iDs on behalf of authors.

We look forward to receiving your revised manuscript.

Kind regards,

Firas H Kobeissy, PhD

Academic Editor

PLOS One

Journal Requirements:

If the reviewer comments include a recommendation to cite specific previously published works, please review and evaluate these publications to determine whether they are relevant and should be cited. There is no requirement to cite these works unless the editor has indicated otherwise.

Please review your reference list to ensure that it is complete and correct. If you have cited papers that have been retracted, please include the rationale for doing so in the manuscript text, or remove these references and replace them with relevant current references. Any changes to the reference list should be mentioned in the rebuttal letter that accompanies your revised manuscript. If you need to cite a retracted article, indicate the article’s retracted status in the References list and also include a citation and full reference for the retraction notice.

[Note: HTML markup is below. Please do not edit.]

Reviewers' comments:

Reviewer's Responses to Questions

**Comments to the Author**

Reviewer #2: All comments have been addressed

Reviewer #3: All comments have been addressed

Reviewer #4: All comments have been addressed

**********

2. Is the manuscript technically sound, and do the data support the conclusions?

Reviewer #2: Yes

Reviewer #3: Partly

Reviewer #4: Yes

**********

3. Has the statistical analysis been performed appropriately and rigorously?

Reviewer #2: Yes

Reviewer #3: Yes

Reviewer #4: Yes

**********

4. Have the authors made all data underlying the findings in their manuscript fully available?

The PLOS Data policy

Reviewer #2: Yes

Reviewer #3: Yes

Reviewer #4: Yes

**********

5. Is the manuscript presented in an intelligible fashion and written in standard English?

Reviewer #2: No

Reviewer #3: Yes

Reviewer #4: Yes

**********

Reviewer #2: This reviewers commends the authors for their extensive revision. The authors have significantly improved the scientific rigor of their study by adding the normal control group as suggested by the previous reviewer, and by expanding the discussion. The only part that requires additional minor revision is a thorough review of the manuscript by preferably a native English speaker to correct the numerous minor grammatical errors.

Reviewer #3: First, the statistical comparisons are unclear. The manuscript should provide clear statistical comparisons between the sham vs. TBI groups and between the sham vs. propranolol-treated TBI groups where applicable. These comparisons are necessary to determine whether TBI significantly alters each measured outcome relative to baseline, and whether propranolol restores the outcome toward sham/control levels. In the figures, please clearly mention sham/control groups and as well as add statistical comparison with Sham/control groups

Second, Figures 2, 3, 4, and 6 do not include scale bars.

the study does not directly demonstrate that the STAT3/MMP2 pathway mediates propranolol’s effects. To support this mechanism, additional functional experiments would be needed. For example, the authors should test whether a STAT3 inhibitor mimics the effect of propranolol after TBI, whether MMP2 inhibition rescues AQP4 localization and glymphatic function, and whether MMP2 overexpression blocks or reduces the beneficial effects of propranolol.

Adding comparison with blocker will make it more strong.

Reviewer #4: 1. The authors have comprehensively addressed the three major issues raised in the previous round. They have added a normal healthy control group to differentiate TBI pathology from drug effects, provided precision and stereotaxic instrumentation details for the glymphatic system detection method, and extensively expanded their discussion to integrate broader clinical frameworks, limitations, and relevant neurological disease models.

2. By incorporating a healthy normal rat control group alongside the TBI and TBI + Propranolol groups, the in vivo experimental structure is now complete and rigorous. The added findings confirm that while propranolol significantly improves spatial learning, memory, and white matter integrity, it does not completely reverse all TBI deficits compared to healthy controls, which demonstrates a realistic and data-supported conclusion. The matching in vitro model using astrocytes treated with norepinephrine further confirms the mechanistic role of the STAT3/MMP2 pathway.

3. The statistical evaluation is robust. An investigator blinded to the experimental groupings conducted the quantification. Multiple brain slices (five slides per sample, eight fields per slide) were digitized uniformly via an objective MCID image analysis system using density thresholds to eliminate bias. Student’s t-test was appropriately utilized to evaluate variations between distinct groups, and data are standardly presented as mean ± SE.

4.The authors state that they have added the missing statistical baselines and control configurations directly into the main text and figures (e.g., Figure 1 revisions) to ensure compliance with PLOS data sharing policies. (Note: Please do a quick final check of the full manuscript PDF to confirm no separate raw data repository was omitted).

5. The revision reads clearly and follows standard scientific English formatting. The expanded text and author replies are organized logically. A minor typographical error exists on Page 2 / Page 6 where the section thickness is written as "80 mm thick sections"—this should be corrected to micrometers (µm) as seen elsewhere in the histology protocol.

**********

what does this mean?). If published, this will include your full peer review and any attached files.

If you choose “no”, your identity will remain anonymous but your review may still be made public.

**Do you want your identity to be public for this peer review?**  For information about this choice, including consent withdrawal, please see our Privacy Policy

Reviewer #2: No

Reviewer #3: No

Reviewer #4: No

**********

[NOTE: If reviewer comments were submitted as an attachment file, they will be attached to this email and accessible via the submission site. Please log into your account, locate the manuscript record, and check for the action link "View Attachments". If this link does not appear, there are no attachment files.]

To ensure your figures meet our technical requirements, please review our figure guidelines: https://journals.plos.org/plosone/s/figures

You may also use PLOS’s free figure tool, NAAS, to help you prepare publication quality figures: https://journals.plos.org/plosone/s/figures#loc-tools-for-figure-preparation.

NAAS will assess whether your figures meet our technical requirements by comparing each figure against our figure specifications.

# Author comment: Author response to Decision Letter 2

**DOI:** 10.1371/journal.pone.0353852.r004

14 Jun 2026

Dear Reviewer:

Thank you for your valuable comments which are very helpful for revising and improving our paper. We have carefully studied your comments and made corresponding revision. Revised portion are marked in red in the submitted paper. The main corrections in the paper and the responds to the reviewer’s comments are as follows:

Reviewer’s comments:

Reviewer #2: This reviewers commends the authors for their extensive revision. The authors have significantly improved the scientific rigor of their study by adding the normal control group as suggested by the previous reviewer, and by expanding the discussion. The only part that requires additional minor revision is a thorough review of the manuscript by preferably a native English speaker to correct the numerous minor grammatical errors.

Reply: Thanks to you for your excellent comment. We tried our best to fix all the grammatical mistakes throughout the manuscript and fully complied with all the reviewer’s requirements.

Reviewer #3: First, the statistical comparisons are unclear. The manuscript should provide clear statistical comparisons between the sham vs. TBI groups and between the sham vs. propranolol-treated TBI groups where applicable. These comparisons are necessary to determine whether TBI significantly alters each measured outcome relative to baseline, and whether propranolol restores the outcome toward sham/control levels. In the figures, please clearly mention sham/control groups and as well as add statistical comparison with Sham/control groups

Second, Figures 2, 3, 4, and 6 do not include scale bars.

the study does not directly demonstrate that the STAT3/MMP2 pathway mediates propranolol’s effects. To support this mechanism, additional functional experiments would be needed. For example, the authors should test whether a STAT3 inhibitor mimics the effect of propranolol after TBI, whether MMP2 inhibition rescues AQP4 localization and glymphatic function, and whether MMP2 overexpression blocks or reduces the beneficial effects of propranolol.

Adding comparison with blocker will make it more strong.

Reply: Thanks to you for your excellent comment. First, all statistically significant comparisons were added to the Results and Figures. Second, scale bars were also added in Figures 2, 3, 4, and 6. Third, we introduced STAT3 and MMP2 inhibitors (S31-201 and SB-3CT) in vivo and vitro experiments. The results as:

Result�

Propranolol improved cognitive function after TBI:

Compared with untreated TBI rats, propranolol, S31-201 and SB-3CT all significantly relieved short- and long-term memory deficits in the novel object recognition and odor tests, though none of the three agents fully recovered memory function compared with the normal rats. In the Morris water maze test, propranolol, S31-201, and SB-3CT all alleviated the deficits in spatial learning and memory in TBI rats. However, none of these three agents fully restored spatial cognitive function compared with the normal rats. (Fig 1)

Figure1. A) The novel object recognition test revealed markedly improved short-term memory in the propranolol, S31-201 and SB-3CT treatment groups compared with the TBI group. Nevertheless, none of the three treatments fully rescued TBI-induced short-term memory deficits compared with normal rats. B) The odor test demonstrated obvious long-term memory improvements in the propranolol, S31-201 and SB-3CT treatment groups compared with the TBI group, whereas complete reversal of long-term memory impairment was not achieved relative to normal rats. C, D) In the Morris water maze test, TBI rats receiving propranolol, S31-201 or SB-3CT displayed significantly ameliorated spatial learning and memory capacities. Still, none of the interventions fully restored spatial cognitive function compared with normal controls. (*p<0.05, n=6 per group)

Propranolol decreased p-tau/Aβ deposition and white matter (WM) damage:

To explore the underlying mechanism by which propranolol improves cognitive function, we examined cerebral p-tau and Aβ deposition levels. The results demonstrated that propranolol, S31-201, and SB-3CT all totally reduced TBI-triggered p-tau and Aβ accumulation in the brain. Moreover, no statistically significant differences were detected among the three treatment groups and the normal group (Figure 2).

To further verify whether propranolol, S31-201, and SB-3CT could rescue TBI-induced white matter (WM) integrity damage, brain myelin density was quantified. As illustrated in Figure 2, myelin density was markedly restored in rats treated with propranolol, S31-201, or SB-3CT relative to untreated TBI rats. Nevertheless, none of the three interventions fully restored myelin density to the level observed in normal rats.

Figure2. (A–F) Propranolol, S31-201, and SB-3CT all totally alleviated TBI-induced cerebral p-tau accumulation. No statistically significant differences were observed among the three treatment groups and the normal control group. G–L) Similarly, propranolol, S31-201, and SB-3CT totally lowered TBI-triggered brain Aβ accumulation, with no significant differences detected among the treatment groups and normal controls. (M–R) Myelin density was significantly recovered in drug-treated rats compared with untreated TBI rats. However, none of the three interventions restored myelin density completely to the level of normal rats. (*p<0.05, n=6/group,10×40 objective lens).

Propranolol recovered glymphatic dysfunction in TBI rats:

Since propranolol was found to reduce cerebral p-tau and Aβ deposition following TBI, we further examined glymphatic system function. The results revealed that propranolol, S31-201, and SB-3CT totally rescued TBI-induced delay in metabolic waste clearance. No significant differences were detected among the three treatment groups and the normal group. (Fig3)

Figure3. (A-F) Propranolol, S31-201 and SB-3CT totally rescued TBI-induced delay in metabolic waste clearance. No significant differences were detected among the three treatment groups and the normal group. (*p<0.05, n=5/group)

Propranolol restored AQP4 expression after TBI:

To elucidate the mechanism by which propranolol ameliorates TBI-induced impairment of glymphatic function, we quantified perivascular AQP4 expression and astrocyte levels in the brain. The results demonstrated that propranolol, S31-201, and SB-3CT totally restored perivascular and astrocytic AQP4 expression relative to untreated TBI rats. No statistically significant differences were identified among the three treatment groups and the normal group. (Fig 4)

Figure4. (A–C, G–I, M–O, S–U, Y–a, e) Propranolol, S31-201, and SB-3CT totally restored perivascular AQP4 (green) expression compared with untreated TBI rats. No statistically significant differences were detected among the three treatment groups and the normal group. Blood vessels were labeled with vWF (von Willebrand factor, red), and cell nuclei were counterstained with DAPI. (D–F, J–L, P–R, V–X, b–d, f) Propranolol, S31-201, and SB-3CT totally restored astrocytic AQP4 (green) expression compared with untreated TBI rats. No statistically significant differences were detected among the three treatment groups and the normal group. Astrocytes were labeled with glial fibrillary acidic protein (GFAP, red), and cell nuclei were counterstained with DAPI. (*p<0.05, n=6/group, 10×40 objective lens)

Propranolol decreased p-STAT3/MMP2 expression after TBI:

To further explore the mechanism of propranolol-induced improvement of cognitive function and glymphatic system recovery after TBI, Western blot was used to test the expression of phosphorylated STAT3 (p-STAT3) and MMP2. The results showed that the levels of p-STAT3 and MMP2 were significantly downregulated in the propranolol-treated TBI rats, but propranolol could not completely reverse the increased levels of p-STAT3 and MMP2 after TBI compared with normal rats. (Fig5)

Propranolol reversed the noradrenaline-mediated increased expression of p-STAT3/MMP2 in astrocytes:

To further clarify the mechanism of propranolol-induced reduction in p-STAT3/MMP2 expression in TBI rats, we treated astrocytes with noradrenaline and propranolol. Western blot data showed that the levels of p-STAT3 and MMP2 were upregulated after astrocytes were incubated with noradrenaline. Subsequent addition of propranolol reversed the noradrenaline-induced elevation in p-STAT3 and MMP2 expression; however, propranolol failed to fully restore p-STAT3 and MMP2 levels to those of the control group. No significant difference was observed between the propranolol-only group and the control group. (Fig5)

Figure5. A, B) MMP2 protein level was markedly decreased in propranolol-treated rats compared with TBI model rats. Nevertheless, propranolol could not fully restore MMP2 upregulation caused by TBI to the level of normal rats. GAPDH was applied as the loading reference. A, C) Western blot quantification demonstrated that p-STAT3 expression was significantly lower in the propranolol intervention group than in the TBI group, whereas complete recovery of TBI-induced abnormal p-STAT3 expression was not achieved by propranolol treatment relative to normal rats. Total STAT3 was set as the loading control. D, E) Incubation with noradrenaline led to significant upregulation of MMP2 in astrocytes. Additional propranolol treatment rescued the noradrenaline-mediated elevation in MMP2 expression, though full normalization to the control group level was not attained. No statistical difference was detected between the single propranolol group and the control group. GAPDH served as the loading control. D, F) Noradrenaline stimulation significantly increased p-STAT3 levels in astrocytes, and this upregulation was partially counteracted by propranolol supplementation. However, p-STAT3 expression could not be fully restored to the baseline level of the control group. No statistical difference was observed between the single propranolol group and the control group. Total STAT3 was used as the loading control. (*p < 0.05)

Propranolol antagonized the noradrenaline-mediated decline expression of AQP4 of astrocytes:

To further elucidate how propranolol elevates AQP4 expression following TBI, astrocytes were subjected to treatments with noradrenaline alone or combined with propranolol, S31-201 or SB-3CT. Immunohistochemical staining was utilized to quantify AQP4 expression in astrocytes. Noradrenaline treatment markedly downregulated AQP4 levels in astrocytes, whereas co-administration of propranolol, S31-201 or SB-3CT fully rescued this noradrenaline-triggered AQP4 downregulation. (Fig6)

Figure6 D–F, S) AQP4 (red) expression was significantly decreased in astrocytes following noradrenaline treatment relative to the control group. G–I, S) Treatment with propranolol fully restored the noradrenaline-evoked downregulation of AQP4 (red) in astrocytes. J–L, S) No statistically significant difference in AQP4 (red) expression was detected between the propranolol single-treatment group and the control group. M–O, S) S31-201 fully rescued the noradrenaline-induced reduction in astrocytic AQP4 (red) expression. P–R, S) SB-3CT also completely reversed the noradrenaline-triggered decline in AQP4 (red) expression in astrocytes. Nuclei were counterstained with DAPI. *p<0.05, 10×20 objective lens, 10 of fields and 6 of cells per field were counted).

Reviewer #4: 1. The authors have comprehensively addressed the three major issues raised in the previous round. They have added a normal healthy control group to differentiate TBI pathology from drug effects, provided precision and stereotaxic instrumentation details for the glymphatic system detection method, and extensively expanded their discussion to integrate broader clinical frameworks, limitations, and relevant neurological disease models.

2. By incorporating a healthy normal rat control group alongside the TBI and TBI + Propranolol groups, the in vivo experimental structure is now complete and rigorous. The added findings confirm that while propranolol significantly improves spatial learning, memory, and white matter integrity, it does not completely reverse all TBI deficits compared to healthy controls, which demonstrates a realistic and data-supported conclusion. The matching in vitro model using astrocytes treated with norepinephrine further confirms the mechanistic role of the STAT3/MMP2 pathway.

3. The statistical evaluation is robust. An investigator blinded to the experimental groupings conducted the quantification. Multiple brain slices (five slides per sample, eight fields per slide) were digitized uniformly via an objective MCID image analysis system using density thresholds to eliminate bias. Student’s t-test was appropriately utilized to evaluate variations between distinct groups, and data are standardly presented as mean ± SE.

4.The authors state that they have added the missing statistical baselines and control configurations directly into the main text and figures (e.g., Figure 1 revisions) to ensure compliance with PLOS data sharing policies. (Note: Please do a quick final check of the full manuscript PDF to confirm no separate raw data repository was omitted).

5. The revision reads clearly and follows standard scientific English formatting. The expanded text and author replies are organized logically. A minor typographical error exists on Page 2 / Page 6 where the section thickness is written as "80 mm thick sections"—this should be corrected to micrometers (µm) as seen elsewhere in the histology protocol.

Reply: Thanks to you for your good comment. The "80 mm thick sections" was corrected to micrometers (µm).

Attachment Submitted filename: Responses_to_reviewer_auresp_2.docx

# Peer review: Decision Letter 2

**DOI:** 10.1371/journal.pone.0353852.r005

**Authors:** Firas Kobeissy

1 Jul 2026

<p>Propranolol Improves Cognitive Function And Glymphatic System Recovery Through STAT3/MMP2 Pathway After TBI

PONE-D-25-47530R2

Dear Dr. yu,

We’re pleased to inform you that your manuscript has been judged scientifically suitable for publication and will be formally accepted for publication once it meets all outstanding technical requirements.

Within one week, you’ll receive an e-mail detailing the required amendments. When these have been addressed, you’ll receive a formal acceptance letter and your manuscript will be scheduled for publication.

An invoice will be generated when your article is formally accepted. Please note, if your institution has a publishing partnership with PLOS and your article meets the relevant criteria, all or part of your publication costs will be covered. Please make sure your user information is up-to-date by logging into Editorial Manager at Editorial Manager® and clicking the ‘Update My Information' link at the top of the page. For questions related to billing, please contact billing support.

If your institution or institutions have a press office, please notify them about your upcoming paper to help maximize its impact. If they’ll be preparing press materials, please inform our press team as soon as possible -- no later than 48 hours after receiving the formal acceptance. Your manuscript will remain under strict press embargo until 2 pm Eastern Time on the date of publication. For more information, please contact onepress@plos.org.

Kind regards,

Firas H Kobeissy, PhD

Academic Editor

PLOS One

Additional Editor Comments (optional):

Reviewers' comments:

Reviewer's Responses to Questions

**Comments to the Author**

Reviewer #3: All comments have been addressed

**********

2. Is the manuscript technically sound, and do the data support the conclusions?

Reviewer #3: Yes

**********

3. Has the statistical analysis been performed appropriately and rigorously?

Reviewer #3: Yes

**********

4. Have the authors made all data underlying the findings in their manuscript fully available?

The PLOS Data policy

Reviewer #3: Yes

**********

5. Is the manuscript presented in an intelligible fashion and written in standard English?

Reviewer #3: Yes

**********

Reviewer #3: (No Response)

**********

what does this mean?). If published, this will include your full peer review and any attached files.

If you choose “no”, your identity will remain anonymous but your review may still be made public.

**Do you want your identity to be public for this peer review?**  For information about this choice, including consent withdrawal, please see our Privacy Policy

Reviewer #3: **Yes:** Pritom Kumar Saha

**********

# Editor report: Acceptance letter

**DOI:** 10.1371/journal.pone.0353852.r006

**Authors:** Firas Kobeissy

PONE-D-25-47530R2

PLOS One

Dear Dr. yu,

I'm pleased to inform you that your manuscript has been deemed suitable for publication in PLOS One. Congratulations! Your manuscript is now being handed over to our production team.

At this stage, our production department will prepare your paper for publication. This includes ensuring the following:

* All references, tables, and figures are properly cited

* All relevant supporting information is included in the manuscript submission,

* There are no issues that prevent the paper from being properly typeset

You will receive further instructions from the production team, including instructions on how to review your proof when it is ready. Please keep in mind that we are working through a large volume of accepted articles, so please give us a few days to review your paper and let you know the next and final steps.

Lastly, if your institution or institutions have a press office, please let them know about your upcoming paper now to help maximize its impact. If they'll be preparing press materials, please inform our press team within the next 48 hours. Your manuscript will remain under strict press embargo until 2 pm Eastern Time on the date of publication. For more information, please contact onepress@plos.org.

You will receive an invoice from PLOS for your publication fee after your manuscript has reached the completed accept phase. If you receive an email requesting payment before acceptance or for any other service, this may be a phishing scheme. Learn how to identify phishing emails and protect your accounts at https://explore.plos.org/phishing.

If we can help with anything else, please email us at customercare@plos.org.

Thank you for submitting your work to PLOS One and supporting open access.

Kind regards,

PLOS ONE Editorial Office Staff

on behalf of

Dr. Firas H Kobeissy

Academic Editor

PLOS One
